# A cross-verified database of notable people, 3500BC-2018AD

**DOI:** 10.1038/s41597-022-01369-4

**Published:** 2022-06-09

**Authors:** Morgane Laouenan, Palaash Bhargava, Jean-Benoît Eyméoud, Olivier Gergaud, Guillaume Plique, Etienne Wasmer

**Affiliations:** 1 CNRS, Université Paris 1 Panthéon-Sorbonne, Centre d’Economie de la Sorbonne, Paris, 75013 France; 2 LIEPP, Sciences Po, Paris, 75007 France; 3 Department of Economics, Columbia University, New York, 10027 USA; 4 KEDGE Business School, Paris, 75012 France; 5 Médialab, Sciences Po, Paris, 75007 France; 6 New York University Abu Dhabi, Social Science Division, Abu Dhabi, 129188 UAE

**Keywords:** Economics, Interdisciplinary studies

## Abstract

A new strand of literature aims at building the most comprehensive and accurate database of notable individuals. We collect a massive amount of data from various editions of Wikipedia and Wikidata. Using deduplication techniques over these partially overlapping sources, we cross-verify each retrieved information. For some variables, Wikipedia adds 15% more information when missing in Wikidata. We find very few errors in the part of the database that contains the most documented individuals but nontrivial error rates in the bottom of the notability distribution, due to sparse information and classification errors or ambiguity. Our strategy results in a cross-verified database of 2.29 million individuals (an elite of 1/43,000 of human being having ever lived), including a third who are not present in the English edition of Wikipedia. Data collection is driven by specific social science questions on gender, economic growth, urban and cultural development. We document an Anglo-Saxon bias present in the English edition of Wikipedia, and document when it matters and when not.

## Abstract (summary)

**Table:** 

Measurement(s)	human biographies
Technology Type(s)	web scraping

## Background & Summary

Since Plutarch’s *Parallel Lives* in the beginning of the second century AD and his 23 biographies, or the Epic of Gilgamesh that further dates back to 2000BC, the task of registering famous individuals and their influence has been a recurrent field of study. It is both a source of fascination for the general public and a resource for scholars as it captures aspects of societies in different centuries. Over the last few years, this task has been undertaken at a much larger scale, with a growing number of databases documenting history, allowing statistical analysis of socio-historical facts, at a scale that had never been reached so far. This approach was pioneered in digital humanities^1^ with a focus on lifetime mobility of 150,000 notable individuals across centuries and continents using all available birth and death data found in Freebase, a Google-owned knowledge database. Freebase and the English edition of Wikipedia was also used to assemble a manually-verified database of 11,341 individuals present in more than 25 language editions of Wikipedia called Pantheon 1.0^2^. For larger databases, the fast-growing verification cost currently prevents scientists from considering in a satisfactory way less prominent individuals, who may however have had a significant impact at a more local level. To overcome these limitations, larger-scale Knowledge Bases^3^ or Wikidata were built automatically from the information retrieved from the Infobox and multiple categories of Wikipedia.

Such projects represent promising developments for the Social Sciences. The database we document here, thanks to its time and spatial coverage and its granularity, is suited to explore gender differences, demography, urban development, cultural transmission, human capital, growth, institutions. See sub-section *Outreach* for an exhaustive list. A good example is a study of the historical development of cities^4^. The authors relate it to the emergence of a creative class using 40,000 individuals, among which 22,000 creatives that are then matched to a sample of 2,200 European cities between 800 and 1800AD^1^. A sample of 300,000 famous people born between Hammurabi’s epoch and 1879, Einstein’s birth year from Index Bio-bibliographicus Notorum Hominum was built to estimate the timing of improvements in longevity and its role on economic growth^5^. The second version of Pantheon (2.0)^6^ also addresses long-run social science trends and offers more statistical power in including around 71,000 with 14+ biographies.

While there has always been a debate regarding the accuracy of Wikipedia as a reliable source in its early years^7^ pointing out more errors than in traditional Encyclopedia, we rejoin other judgements that the information present is highly reliable. In fact, the main problem arises from omissions^8^, that will naturally decrease as Wikipedia expands. Our dataset must be seen as an evolving object. Our contribution is primarily a methodological one, how to make use of the information available and as accurately as possible. Our next goal is to build a comprehensive and accurate Knowledge Base of notable individuals for immediate use in the social sciences and in particular econometric and statistical analyses. It complements existing approaches in several ways. First, we collect a massive amount of data that leads to several cross-verifications. It is based on multiple sources (various Wikipedia editions and Wikidata) and deduplication techniques. Combining Wikipedia and Wikidata brings 2.72% new birth dates, 8.16% new occupations and 17.16% new citizenships. We find that there are a limited number of errors in the part of the database that contains the most documented individuals. We also find non trivial error rates (around 1%) in the bottom part of the notability distribution, due to sparse information and classification errors or ambiguity. This either requires manual corrections for future use or an appropriate statistical treatment of these errors in statistical approaches. Combining Wikipedia and Wikidata allows to correct about 0.5% of all detected errors. We build and make accessible a database with a limited number of errors. The scope of the final database does not cover all Wikipedia, but only when the content could be cross-verified between different language editions and Wikidata. Second, data collection is driven by specific social science questions on gender, economic, growth, urban and cultural development. We document the Anglo-Saxon bias naturally present in existing projects based on the English edition of Wikipedia.

This strategy results in a cross-verified database of 2.29 million unique individuals (an elite of 1/43,000 of human being having ever lived) among which 30% come from the 6 non-English editions of Wikipedia, a significant improvement over earlier works that have only focused on English Wikipedia only. We verified our algorithms with 5,000 manual checks. Error rates vary by sources and notability levels. We therefore recommend to limit the database to individuals with at least two sources and not to use an exhaustive database of 4.7 million people. The large number of individuals in the database is the main distinctive feature of our work. Lesser-known individuals may have contributed in a significant way to the social, economic or cultural development of their community (city, region or country) in fostering innovation and growth in their cluster. To accurately measure the relative influence of each individual present in our database, we build a notability index extending earlier work^2^ and allowing to study different groups of the distribution of notability. Note that the ranking should not be used to compare pairs of individuals but rather to illustrate the differences in demographics or sectors across quantiles of notability.

## Methods

### Universe described

Social scientists are interested in significant people insofar as they have influenced the trajectory of the society in their times or later on. Let us call ${\mathfrak{S}}$ the universe of these significant individuals, without any further discussion of what influence means - it is actually entirely specific to the question social scientists would ask, e.g. the dynamics of the arts, or science, or demography, etc. Let us call ${\mathfrak{D}}$ the universe of already detected individuals, for instance all those individuals above a certain visibility threshold at their time, that would be invariant over time, e.g. the so called ‘elite’, the top famous individuals. Some of them will be part of our data collection process, from Wikipedia and Wikidata (let us call this set ${\mathfrak{W}}$), while others are in known sources but unexploited yet, or not yet in the Wiki universe (let us denote it ${\mathfrak{U}}$). Some individuals may have been significant and even remembered for a while, but are currently forgotten. We can call this set ${\mathfrak{F}}$. To sum up, the theoretical universe of significant individuals is decomposed as:

$${\mathfrak{S}}={\mathfrak{F}}\cup {\mathfrak{D}}={\mathfrak{F}}\cup {\mathfrak{W}}\cup {\mathfrak{U}}$$

We try to reach ${\mathfrak{S}}$ by ${\mathfrak{w}}$ with automatic routines. In the future, unexploited archives in ${\mathfrak{U}}$ will gradually converge to the Wiki universe ${\mathfrak{W}}$, while forgotten individuals in ${\mathfrak{F}}$ will - albeit slowly - be gradually rediscovered by historians, or scholars in the arts or science disciplines. Therefore, further editions of this database will cover more individuals as more information will become available in Wikipedia and Wikidata in the future.

### A social science background

Social scientists develop models to illustrate causal mechanisms. As a result, we need to collect data in a way that fits the typology of these models. To encompass a wide range of human activities and domains of influence, we follow the seminal work on the so-called Baumol’s cost disease^9^. This refers to a situation in which a sector without productivity gains (such as performing arts until the beginning of the XXth century) faces higher wages due to productivity gains in other sectors. We assume that the amount of global wealth produced by humans in a given year is of two types, ${Y}^{M},{Y}^{I}$, where ${Y}^{M}$ is the amount of materialistic goods and services generated by the commonly-called productive sector, and ${Y}^{I}$ is the amount of non-materialistic goods and services created in other sectors of the economy such as arts, leisure, health, sports, etc. The evolution of ${Y}^{M}$, unlike ${Y}^{I}$ mostly made of labor-intensive activities, will benefit from science innovations and technological progress: the famous Magic flute by Wolfgang Amadeus Mozart still requires the same amount of resources (time, performers, space, etc.) nowadays as in 1791, when it was first performed. Absent any change in preferences, the Baumol effect states that the size of the second sector should constantly decrease over time. However, the first sector produces essential goods where the second sector produces luxury goods, implying that the relative demand for the arts would actually increase and this prevents the arts sector from continuously declining and eventually disappear.

Additional notations can help visualize this. Materialistic goods and services are produced by combining a set of inputs ${\mathscr{B}}$ featuring the economy and business, which, for simplicity, include labor and capital. The level of total factor productivity ${\mathscr{A}}$ combines the various outputs of science, education, technology and discoveries of all kinds denoted by ${\mathscr{B}}$:

1 $${Y}^{M}={\mathscr{A}}f({\mathscr{B}}).$$

while the second component ${Y}^{I}$ mostly depends on the number of famous individuals in creative activities. Last, the consumption vector $\overrightarrow{{\mathscr{C}}}=({C}^{M},\,{C}^{I}{)}^{{\rm{{\prime} }}}$ is only a fraction $(1-\tau )$ of the amount of global wealth as follows:

2 $$\overrightarrow{{\mathscr{C}}}=\overrightarrow{{\mathscr{C}}}[(1-\tau )({Y}^{M}+{Y}^{I})],$$

where $(1-\tau )$ is typically determined by the way a society is governed by politicians, military, religious authorities, noble people, and *τ* is an administration tax, featuring more or less efficient societies and resources diversion^10^. The demand function $\overrightarrow{{\mathscr{C}}}$ is such that the first dimension (*M*) is a necessity good and the second dimension (*I*) gets a higher demand as income and wealth grow. This framework will help us understand long-run trends in the relative importance of different fields among notable individuals: each broad occupation defined later in the data will be associated with one of the theoretical concepts from the paragraphs above.

### Preview of the data

The database is organized as follows: each row corresponds to one individual, with a name and the unique identifier (section *Extracting raw data from* Wikidata *and* Wikipedia), the birth date and gender (section *Demographics: birth, death and gender*), an imputation if missing, a range if known from a decade or century, up to two fields called domains of influence and an estimated weight for each domain (section *Domains of influence and occupations*), birthplace and death place, longitude and latitude (section *Data records*), a proxy for citizenship (section *Citizenship*) and a notability index calculated from 5 variables (section *Measuring notability*). The sample choice is described in section *Extracting biographic information from a restricted sample*.

### Extracting raw data from Wikidata and Wikipedia

To build the most comprehensive, reliable database about notable individuals, and organize it in a way that can be later used by social scientists, we consider two main sources of information: information from Wikidata and information contained in 7 language editions of Wikipedia. We extract key information on humans present either in one or both sources, and then merge this information to avoid 1) dealing with duplicate individuals and 2) cross-verify the information contained in both universes.

#### Wikipedia

This paper uses a bottom-up procedure based on Wikipedia categories. Categories are commonly used in Wikipedia to link articles under a common topic and are found at the bottom of the page. All existing categories about individuals were systematically analyzed and retrieved by scraping. Using the relevant urls corresponding to human biographies, we scraped the 7 following Wikipedia language editions: English, French, German, Italian, Spanish, Portuguese and Swedish that we call hereafter the *European editions*. Within the sample of individuals with at least one biography in the 7 language editions, 52.8% have 1 biography, 18.1% have 2 biographies (cumulative 70.9%), 8.7% have 3 biographies (cumulative 79.6%), 5% have 4 biographies (cumulative 87.8%) and 12.2% have 5 biographies or more. See Appendix A for a detailed description of the method.

#### Wikidata

In the Wikidata universe, we use the “instance of humans” Q5 category to define our sample of individuals. Information such as first name, family name, gender, country of citizenship, date of birth, date of death (if applicable), domain of influence, as well as the Q code (identifier) of the individual are retrieved. In addition to this first set of variables, we also extract the exhaustive list of Wikipedia urls contained in each Wikidata entry, to deal with multiple biographies as explained below. We also collect the sources behind the Wikidata page, used in Figure S7 in Appendix.

### Merging Wikidata and Wikipedia

Wikidata and Wikipedia contradict each other on key information about notable individuals. Considering 7 different Wikipedia editions, instead of one, naturally increases that heterogeneity. In this paper, we use this feature to improve the reliability of each information extracted from both repositories. To do so, we develop and use in what follows a series of algorithms to i) come up with a relevant sample of humans, ii) eliminate duplicate biographies, iii) detect systematic errors contained either in Wikipedia or Wikidata and correct them.

#### Humans and non-humans

A large number of entries are wrongly identified as humans in Wikidata because they contain the Q5 code which stands for ‘instance of humans’ but some may not be humans. The same issue arises in Wikipedia: there are non-humans despite being identified as humans. These entries are either biblical, mythological, fictional characters, animals, music bands, events, murder cases, etc. We use a list of expressions such as “murder of”, “list of”, “duos”, “bands”, “attack of”, etc. to detect such cases. Let us illustrate the difficulty with a few examples. https://www.wikidata.org/wiki/Q656193 is an example of a page of a real human in the Czech edition of Wikipedia linked to fictional characters, yet as of the time of data extraction, wrongly classified as a human. A slightly more ambiguous case is that of https://www.wikidata.org/wiki/Q1278485, a possibly fictional character subject of a story in Ancient Rome - and in any event the pages describe the person as a fictional character, while she is classified as human in Wikidata. In total, we identify 20,000 entries corresponding to 16,000 questionable “pseudo-individuals” that we eliminate from our database.

#### Dealing with multiple biographies

By construction, a given individual has a maximum of eight observations in the database once merged: 7 Wikipedia biographies plus one Wikidata entry. There are several cases to consider to eliminate duplicate observations from this universe of around 6.3 million biographies/entries. The most common situation is one individual present in Wikidata and in at least one of the 7 language editions of Wikipedia with one unique identifier present in both universes. In such situations, we eliminate duplicates by checking whether i) the links present in the Wikipedia section of each Wikidata biography correspond to each other and ii) the Wikidata links retrieved from the “Tools” section of each Wikipedia biography correspond to each other; there are indeed sometimes inconsistencies between these sources. We wrote a batch file to manually modify about 2000 Wikidata identifiers out of 4.7 millions not available at the time of scraping.

#### Treating duplicates

In addition, a given individual may have more than one biography either a) within the same Wikipedia edition or b) across different editions without any explicit link between these duplicate biographies or c) in Wikidata and/or Wikipedia under different Q codes (identifiers), due to the large number of contributors. Some of them may fail to detect or ignore the work of others. As an example, Sarendy Vong has two separate biographies in the English edition: https://en.Wikipedia.org/wiki/Sarendy_Vong and https://en.Wikipedia.org/wiki/Vong_Sarendy. Appendix A.2 provides further details about this methodology. We eventually removed 0.7% of individuals (34,562/4,678,040). Although we cannot claim we eliminated all duplicates, we adopted a conservative approach in multiplying the number of tests we have run and rather eliminated false duplicates than keeping true duplicates.

### Exhaustive sample: descriptive statistics

#### Wikipedia: comparing language editions

The 5 most popular language editions in Wikipedia in number of biographies are, in decreasing order, 1) English, 2) German, 3) Japanese, 4) French and 5) Russian. Considering the German edition after English adds 340,913 individuals (of which 259,013 individuals have a unique biography in German). It is worth noting here that these 340,913 individuals would have been ignored without considering the German edition. In the exhaustive sample, the Japanese edition brings 179,466 new individuals, the French edition adds 155,391 individuals, the Russian edition adds 156,728 individuals. The full list of incremental additions is available in Table S2 in Appendix A.5 and provides a better sense of the relative importance of each language edition.

#### Main sample characteristics

Overall, we gather information about 4,678,040 distinct and unique notable individuals out of their 6,291,767 multiple biographies extracted from 7 different Wikipedia language editions and Wikidata. There are 2,291,817 individuals (49%) with at least one biography in Wikipedia along with a Wikidata entry. In addition, there are 2,386,223 notable individuals (51%) with a Wikidata entry but no existence in the 7 European Wikipedia language editions.

Table 1 collapses these famous individuals in 6 groups to help us figure out the respective contribution of both Wikidata and Wikipedia. In subsequent columns, we display similar statistics for different individual profiles according to their popularity in Wikipedia, in particular those with at least one biography among the 7 European language editions (described above) and at least 14 language editions (Pantheon 2.0). Each row corresponds to a specific language group, not based on a linguistic classification but mostly on geography to capture the socioeconomic groups formed around empires and trade areas: *English* (individuals with at least one biography in the English edition), *Western* (individuals with one biography in a language part of the Western world but not English), *Eastern* (individuals with an edition that is part of the eastern world but not English/Western), *Eurasia-Arabia* (individuals with an edition that is part of the Eurasia-Arabia world but not English/Western/Eastern), *Southern and natives* (individuals with an edition that is part of the Southern/natives world but not English/Western/Eastern/Eurasia-Arabia), and Wikidata only (individuals with one Wikidata entry but no existence in the Wikipedia universe). Table S2 in Appendix shows the diversity of languages and the number of *new* individuals brought by language editions. However, we will only consider those individuals from pages we could analyze, e.g. wih a biography in one at least of the 7 language editions analyzed.

**Table 1:** Marginal contribution of different groups of Wikipedia language editions.

	All Wikipedia & Wikidata		At least one European edition		At least 14 editions	
	Freq.	%	Freq.	%	Freq.	%
	Exhaustive sample		Restricted sample		Pantheon 2.0	
Wikipedia *editions (by recursive language groups)*						
English (En)	1,578,917	33.8	**1,547,174**	67.5	76,139	99.7
Western (We)	1,327,543	28.4	**744,643**	32.5	240	0.3
Eastern (Ea)	342,783	7.3	0	0.0	0	0.0
Eurasia - Arabia (EuAr)	223,213	4.8	0	0.0	0	0.0
Southern & natives (Sn)	4,249	0.1	0	0.0	0	0.0
Wikidata *only*	1,201,335	25.7	0	0.0	0	0.0
Total	4,678,040	100	**2,291,817**	100	76,379	100

Notes. English (*En*): individuals present in the English edition; Western (*We*): individuals absent from the *En* edition but present in *We* editions; Eastern (*Ea*): individuals absent from the *En* & *We* groups but with at least one biography in editions of the *Ea* group; Eurasia-Asia (*EuAr*): individuals absent from the previous groups (*En*, *We*, *Ea*) but present in at least one *EuAr* edition. Southern & natives (*Sn*): individuals absent from the other groups (*En*, *We*, *Ea*, *EuAr*) but present in at least one edition of the *Sn* group. Wikidata *only* includes individuals with a Wikidata biography only. In columns, we display the total headcount figures (number and share) in various samples: all individuals regardless of their Wikipedia profile (exhaustive sample); individuals with at least 1 biography among the 7 European editions; individuals with 14+ biographies in the Wikipedia universe.

The exhaustive sample (Table 1, column 1) contains 1,578,917 individuals in the first group (English), 1,327,543 in the second group (Western non-English). The third, fourth and fifth groups correspond to 342,783; 223,213 and 4,249 notables from the Eastern, Eurasia-Arabia and Southern/natives worlds respectively. The last group which is made of individuals with one Wikidata entry only represents around 1/4th of all individuals.

The information present in existing databases are based on the English edition of Wikipedia only^1,2^. This project, by considering other language editions and Wikidata, almost triples the universe of notable individuals as follows: Western languages (+27.0%), Eastern languages (+7.3%), Eurasian-Asian languages (+4.8%) and Southern & native languages (0.1%), Wikidata (+25.7%). Considering 6 additional language editions results in 3,099,123 newly detected in the exhaustive sample.

### Measuring notability

Many individuals have low visibility and sparse information. To disentangle the most visible from the less visible, we build a synthetic notability index using five dimensions to figure out a ranking for this broader set of individuals. These dimensions are:

1. the number of Wikipedia editions of each individual;
2. the length, i.e total number of words found in all available biographies. It is equal to zero for individuals with just one Wikidata entry and no biography in Wikipedia;
3. the average number of biography views (hits) for each individual between 2015 and 2018 in all available language editions, using an API available in https://wikitech.wikimedia.org/wiki/Analytics/AQS/Pageviews or zero in the absence of a Wikipedia biography;
4. the number of non-missing items retrieved from Wikipedia or Wikidata for birth date, gender and domain of influence. The intuition here is that the more notable the individual, the more documented his/her biographies will be;
5. the total number of external links (sources, references, etc.) from Wikidata.

We then determine the quantile values from each dimension and add them all to define our notability measure that is used to compare/rank individuals over time and across space. Arguably, it is harder to compare individuals born in different centuries and we will not present any of these comparisons, since it tends to favor individuals born recently^2^. Alternative choices for the weights of the sub-components can be made depending on the question that is tackled. We also reiterate that even within centuries or within domains of influence, pairwise comparisons make little sense and the notability index is more of a weight to statistically assess the influence or identify large groups of influence (e.g. by quintile of notability index). This being said, Table 2 shows the 10 most renowned individuals per period and gender. Examples of the most famous individuals present in the English edition are Barack Obama, Cristiano Ronaldo or Albert Einstein. Figure S5 in Appendix displays a cloud of the top 3000 individuals and suggests biases in gender (very few women), fields (large representation of culture and notability against for instance business) and historical periods (contemporaneity bias) that we address next. Other language groups naturally include lesser-known individuals like French film director Olivier Nakache (*Western*), Japanese actor Ryō Iwamatsu (*Eastern*), scientist Qayum Nasıyri (*Eurasia-Arabia*), former writer Boerneef (*Southern & natives*) and art historian Martin Hardie present in Wikidata *only*. Table S3 in Appendix shows the 5 most famous individuals in each recursive language group. These individuals have achieved a lower level of international recognition but may have had a significant impact at the local level, in their own country, region or city. In the rest of the paper, we explore how these “newly added” individuals differ from those present in the English edition on a few dimensions such as year of birth/death, gender, occupation or citizenship. In particular, the Section Historical trends will systematically show the contribution of different samples (e.g. Fig. 3 or the right part of Fig. 4 and subsequent Figures).

**Table 2:** Most famous historical figures: breakdown by period of death - or contemporaneous - and by gender.

Death (AD)	*Women*
Before 500	Catherine of Alexandria, Saint Cecilia, Cleopatra, Helena (empress), Hypatia, Livia, Saint Lucy, Messalina, Sappho, Agrippina the Younger
501–1500	Eleanor of Aquitaine, Joan of Arc, Clare of Assisi, Hildegard of Bingen, Mary of Burgundy, Isabella of France, Elizabeth of Hungary, Murasaki Shikibu, Catherine of Siena, Bridget of Sweden
1501–1750	Anne, Queen of Great Britain, Catherine of Aragon, Anne Boleyn, Elizabeth I of England, Lady Jane Grey, Isabella I of Castile, Mary I of England, Mary, Queen of Scots, Catherine de’ Medici, Teresa of Ávila
1751–1900	Marie Antoinette, Jane Austen, Emily Dickinson, Catherine the Great, Thérèse of Lisieux, Ada Lovelace, George Sand, Mary Shelley, Maria Theresa, Mary Wollstonecraft
1901–1979	Agatha Christie, Marie Curie, Anne Frank, Judy Garland, Frida Kahlo, Marilyn Monroe, Florence Nightingale, Édith Piaf, Queen Victoria, Virginia Woolf
1980–2019	Diana, Princess of Wales, Marlene Dietrich, Audrey Hepburn, Katharine Hepburn, Whitney Houston, Grace Kelly, Elizabeth Taylor, Mother Teresa, Margaret Thatcher, Amy Winehouse
Alive	Beyoncé, Hillary Clinton, Lady Gaga, Selena Gomez, Angelina Jolie, Madonna (entertainer), Rihanna, J. K. Rowling, Britney Spears, Meryl Streep
**Death (AD)**	***Men***
Before 500	Paul the Apostle, Aristotle, Marcus Aurelius, Julius Caesar, Cicero, Alexander the Great, Augustine of Hippo, Jesus, Plato, Socrates
501–1500	Dante Alighieri, Thomas Aquinas, Francis of Assisi, Avicenna, Charlemagne, Genghis Khan, Muhammad, Petrarch, Marco Polo, Rumi
1501–1750	Johann Sebastian Bach, Christopher Columbus, René Descartes, Galileo Galilei, Henry VIII of England, Martin Luther, Michelangelo, Isaac Newton, William Shakespeare, Leonardo da Vinci
1751–1900	Ludwig van Beethoven, Charles Darwin, Vincent van Gogh, Victor Hugo, Abraham Lincoln, Karl Marx, Wolfgang Amadeus Mozart, Napoleon, Friedrich Nietzsche, George Washington
1901–1979	Charlie Chaplin, Winston Churchill, Albert Einstein, Sigmund Freud, Mahatma Gandhi, Che Guevara, Adolf Hitler, John Lennon, Pablo Picasso, Elvis Presley
1980–2019	Muhammad Ali, David Bowie, Fidel Castro, Salvador Dalí, Stephen Hawking, Michael Jackson, Nelson Mandela, Ronald Reagan, Frank Sinatra, Andy Warhol
Alive	Woody Allen, George W. Bush, Bill Clinton, Bob Dylan, Eminem, Paul McCartney, Lionel Messi, Barack Obama, Arnold Schwarzenegger, Donald Trump

Notes. 10 most famous historical figures by period (of death year or contemporaneous) and gender. The ranking is computed from a notability index described in the text.

### Extracting biographic information from a restricted sample

As reported in Appendix Section B.3, information about lesser-known individuals is quite often sparse and does not allow to apply our cross-verification technique in a satisfactory way to detect systematic errors contained either in Wikipedia or in Wikidata and correct them. This is the reason why we decided to focus on a sub-sample of individuals with at least one biography among the 7 European language editions of Wikipedia we selected.

We use this sample to highlight the difference between the English edition (used in existing projects) and the other editions and document a potential Anglo-Saxon bias. As displayed in column 2 of Table 1, this sample contains information about 2,291,817 notable individuals with at least one biography among the universe of Wikipedia editions we considered (7 European language editions enumerated above) plus a Wikidata entry.

#### Demographics: birth, death and gender

We consider all personal (he/she) and possessive (his/her) pronouns present in the first part of each available Wikipedia biography to figure out a gender for each individual. In case both masculine and feminine pronouns are detected, we use the one that appears first in the biography to determine the individual’s gender. We then compared it to the one extracted from Wikidata, when reported. A fraction of 0.03% of the restricted database has an “Other” gender (609 individuals), corresponding mostly to transgender persons (e.g. https://www.wikidata.org/wiki/Q57585712) or non-binary persons (e.g. https://www.wikidata.org/wiki/Q55237966).

We keep the gender information present in Wikidata when both sources contradict each other. In case the information is missing in one universe, we use the gender found in the other source when available.

When the birth and death information is present in both sources, we first determine the most recurring year among the different available (up to 7) language editions of Wikipedia, and compare it with the year extracted from Wikidata when reported. When there is a conflict between different sources, we give priority to Wikidata. See Appendix A6 for details. There are a few cases of super centenarians, allegedly known as having lived more than 120 years. The only case where we deleted the birth date is Trailanga (1607–1887).

We next calculate the number of living individuals in a given year in the database. This is represented in Appendix Figure S3. There is an exponential evolution of the sample size over time. The ratio of the number of notable individuals in the restricted sample over the world population (from two different estimates^11,12^) is not constant: as illustrated in Appendix Figure S2, the ratio of the number of famous people alive at a given point in time to the world population increased from 500BC to 1950. The ratio was around 1 over 250,000 in the Antiquity and is 1 over 3,000 in 1950. Under the assumption that the fraction of “famous individuals” is constant throughout history, one can calculate the rate at which these were forgotten. Details of the calculation can be found in Appendix A.4. Under the assumption that people are forgotten at rate *b*, the fraction of famous people forgotten relative to total population is $1-{(1-b)}^{T}$ where *T* is distance to present. The value of  b is estimated in Appendix and corresponds to 15.2% each century, or 56.1% after 500 years, or 80.8 % after 1000 years. Therefore, it is likely that a large number of individuals who should have been remembered given their achievement are currently not listed in the database, again under the assumption that the detection threshold of the recent period should have been applied since the start of our sample. In Appendix Figures S3 and S4, we show the time evolution of the sample size and its distribution across the English, Western, Eastern, Eurasia-Arabia, Southern & natives language groups from the exhaustive dataset.

#### Domains of influence and occupations

We proceed the same way as for dates, and systematically compare the information collected from Wikipedia and Wikidata to figure out the domain of influence, the sector of activity or the field where notable individuals are meant to have had an impact. The list of occupations is reported below. The choice of the categories is based on the theory representation developed in the above Section “A social science framework”, differentiating producers of material/commercial goods and services, the producers of ideas or art, and the producers of administration services, security and rules. The choices turn out to be largely consistent with other typologies for most occupations^2^ and culture^13^. The “Other” section regroups individuals whose notability originates from i) a direct link with notable individuals e.g, the children of a ruler who themselves did not rule) ii) an occupation that is not classified in culture (e.g. librarian) or iii) only for their negative notorious acts (e.g. criminal, serial killer). When multiple occupations can be extracted from a maximum of 7 sources, we adopt a frequentist approach, using the mode and the frequencies of competing occupations to select a main one and sometimes a secondary one, as discussed in Appendix B.1.

- **Discovery/Science** *(contributes mostly to total factor productivity* ${\mathscr{A}}$*)*:
  - Academia (Research, Historian, Physician, Scientist, Academic, etc.)
  - Explorer (Engineer, Explorer, Inventor, Sailor, Pioneer, etc.)

- **Culture** *(contributes mostly to immaterial goods and services* ${Y}^{I}$*)*
  - Core (Actor, Writer, Painter, Singer, Music, etc.)
  - Periphery (Journalist, Architect, Model, Designer, Presenter, etc.) *(may also contribute to business/econ inputs* ${\mathscr{B}}$*)*

- **Leadership** *(contributes mostly to the organization of society and reduce the administration cost τ) or raise it through rent-seeking activities*
  - Politics (Politician, Activist, Revolutionary, Trade unionist, Minister, etc.)
  - Military (Military, Officer, Commander, Soldier, Army, etc.)
  - Law (Lawyer, Diplomat, Judge, Jurist, Civil service)
  - Nobility (Aristocrat, Noble, King, Sovereign, Monarch, etc.)
  - Religious (Priest, Prelate, Rabbi, Missionary, Bishop, etc.)
  - Corporate Leadership (Business, Entrepreneur, Bank, Merchant, Manager, etc.) *(may also contribute to* ${\mathscr{B}}$*)*

- **Sports/Games** (Football, Player, Sport, Baseball, Basket, etc.) *(contributes mostly to* ${Y}^{I}$ *but also to* ${\mathscr{B}}$*)*
- **Other**
  - Worker (Farmer, Librarian, Musher, Bookseller, Printer, etc.) *(contribute mostly to* ${\mathscr{B}}$*)*
  - Family (Son, Daughter, Child, Wife of, Father, etc.)
  - Misc. (Esperantist, Criminal, Convict, Killer, Philanthropist, etc.)

**Fig. 1:**
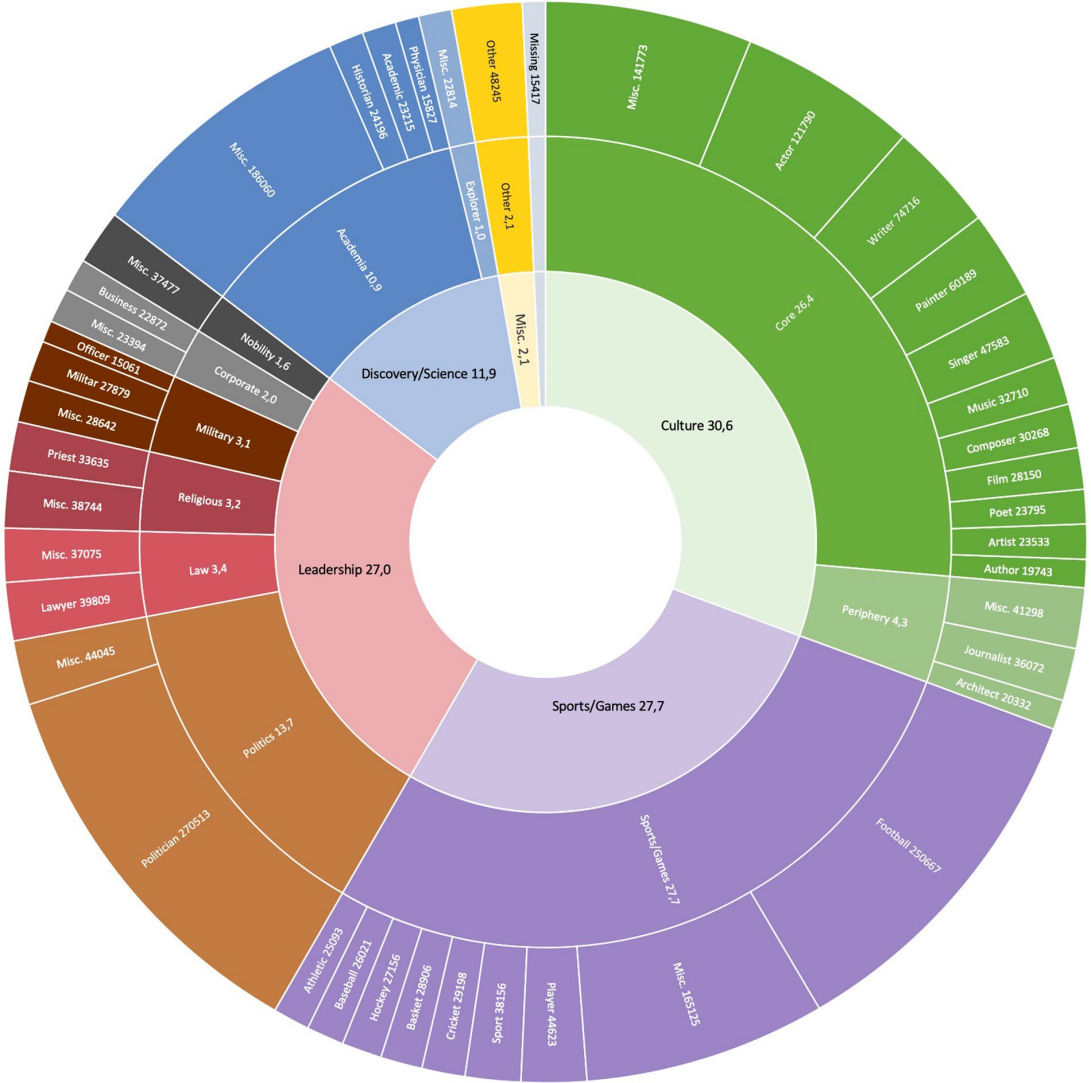
Sunburst: relative importance of the main occupations and domains of influence. Notes. Cross-verified, restricted database (at least one Wikipedia edition among the 7 European languages analyzed), see Section *Extracting biographic information from a restricted sample*. The occupations are defined in Section *Domains of influence and occupations*.

Figure 1 describes the relative importance of domains of influence in the restricted sample described above. Interestingly, the four most popular domains are, in decreasing order: Culture (30.6%), Sports/Games (27.7%), Leadership (27.0%), Discovery/Science (11.9%) and Other (2.1%). People that are most remembered in Wikipedia have specialized in sectors producing non-materialistic goods and services and thus contribute to ${Y}^{I}$ and to a much lower extent to ${Y}^{M}$. In the Culture category, the vast majority of individuals with a biography had or still have a career in one of the many core cultural industries (26.4%) and more sporadically in the peripheral cultural industries (4.2%). The Leadership category is dominated by Politicians (14.2%), Military (3.2%), Lawyers (3.2%), Religious faces (3.1%), Corporate leaders (2%) and Nobles (1.7%). The academic sector (11.1%) dominates the Discovery/Science category. Explorers correspond to a minor part of this category (1.1%).

**Fig. 2:**
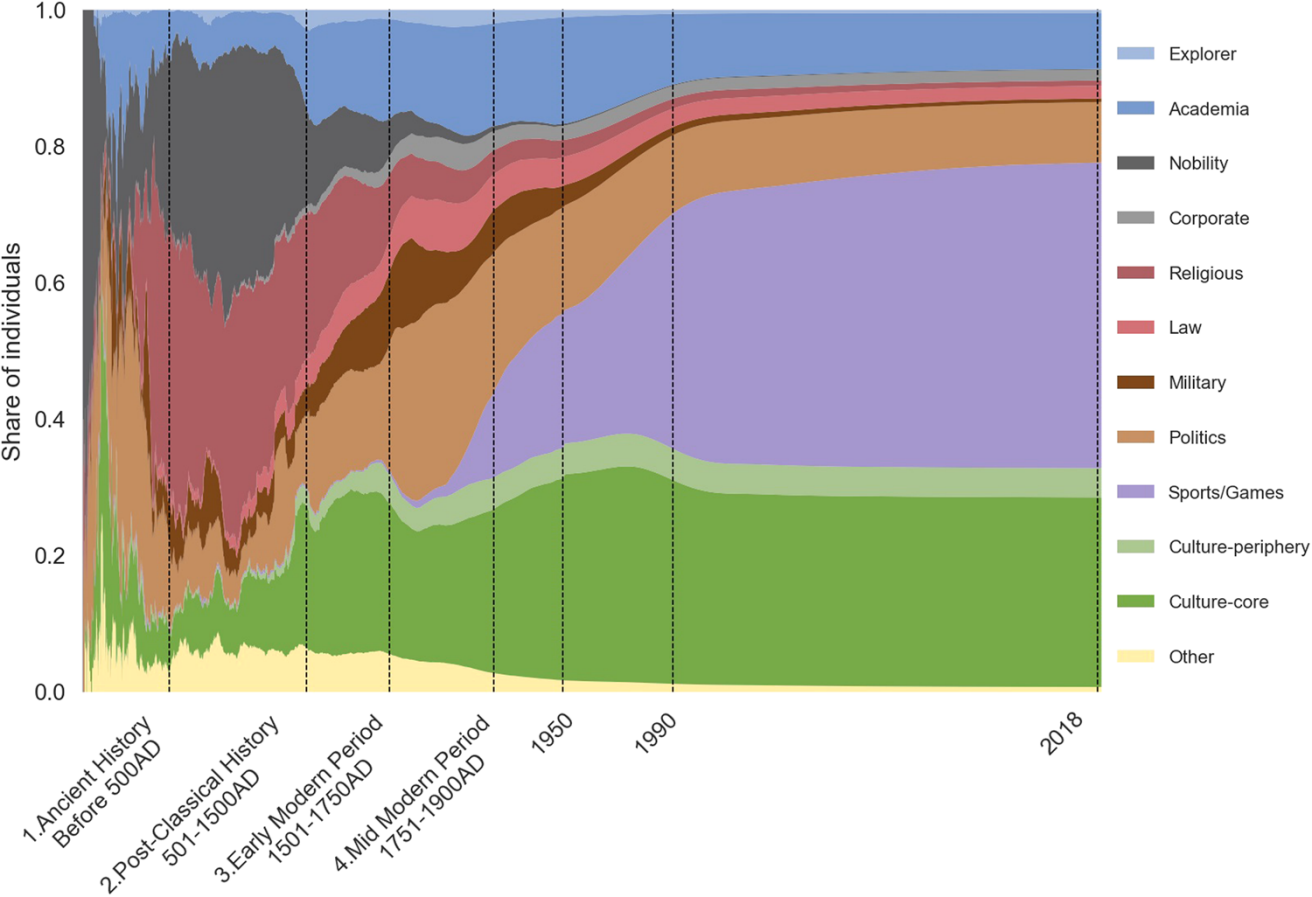
Share of individuals present in the database, breakdown by domain of influence. Notes. Cross-verified, restricted sample (at least one Wikipedia edition among the 7 European languages analyzed), see Section *Extracting biographic information from a restricted sample*. For a given year, the number of living individuals is calculated by summing up all individuals such that $birth\_date\le year\le death\_date$. When not available, the date of birth (resp. death) is estimated from the estimated average longevity over the period. The following log transformation has been applied to the time axis: $year\to 8.5-log(2019-year)$.

Figure 2 shows the time evolution of the share of these different domains. We clearly see on this graph the prominent role played by religious figures and the military until the beginning of the 15th century, a slow rise in Academia, Politics and Culture afterwards, and the rapid rise of a new entertainment category, Sport and Game over the 20th century that even become dominant after 1950. This trend corresponds to another trend, the secular rise in leisure and the decline in hours worked over the last two centuries^14^ due to a combination of technical progress sustaining living standards and leisure and many other immaterial goods being luxury goods.

#### Citizenship

For citizenships, we collect and then compare the information available from both Wikipedia and Wikidata. From Wikipedia, we retrieve up to two citizenships per language edition, based on the current name of the country therefore summing to a maximum of 14 possible citizenships. We keep the two most frequent citizenships of this pool and compare them to Wikidata. The comparison group from Wikidata retains a list of up to 10 citizenships. We group these citizenships into meaningful entities that are not necessarily time-invariant: e.g. entities such as “Allemagne”, “Berlin”, etc. are clubbed under Germany, “Ting Dynasty”, “Ming Dynasty”, “Song Dynasty”, etc. are clubbed under Old regimes in China, “Russian Empire”, “Tsardom of Russia”, “Grand Duchy of Moscow”, etc. are clubbed under Old Russia, “First French Republic”, “Second French Republic”, “Third French Republic”, etc. are clubbed under France. For individuals living in the modern state, we retain the name of the modern entity, for e.g. for a person who lives or have lived under today’s People’s Republic of China, the reported citizenship is simply China. We also keep three empires, Holy Roman Empire, Roman Empire and Soviet Union in these aggregate groups. We skipped extremely unfrequent citizenships such as Chenla Kingdom, Sultanate of Hobyo, Lordship of Carpi etc. that were not aggregated with our main citizenship (0.004% of those with a citizenship only).

In 95% of cases, Wikidata and Wikipedia gave the same citizenship. When they instead contradict each other, we adopt a decision rule detailed in Appendix B.2 giving higher priority to information present in the Infoboxes from Wikipedia and Wikidata. To classify individuals under the old regimes v.s. the current state, we exploit the information on acquisition of sovereignty of modern state. We use information available on the collapse of empires such as the Holy Roman Empire or the Soviet Union to correctly classify individuals into these supranational entities. Finally, a fraction of individuals may have several citizenships and we report two of them if relevant.

### Historical trends

Additional editions we analyzed beyond English provide a broader and finer coverage of some key periods in human history and social trends. We review here only a small number of examples of interest for the sake of conciseness. With a few emblematic historical events in mind, we ask whether the database could quantitatively illustrate these periods.

#### From Dark Ages to the Modern age: the rise of notable women

**Fig. 3:**
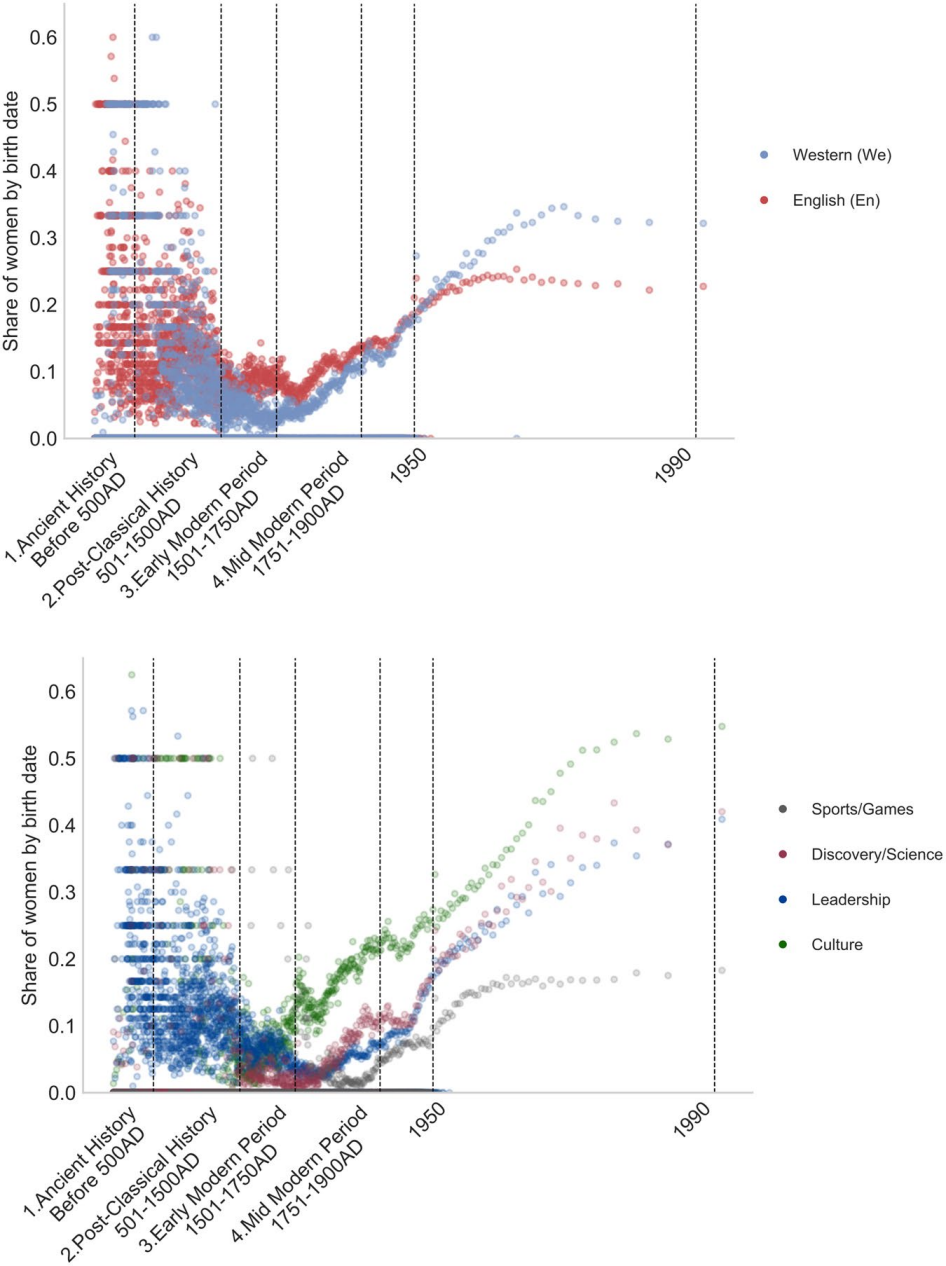
Share of women (top, by continent, bottom, by domain), 1000BC-2000AD. Notes. Cross-verified, restricted sample (at least one Wikipedia edition among the 7 European languages analyzed), see Section *Extracting biographic information from a restricted sample*. Western refers to Western, non-English editions of Wikipedia analyzed. The occupations are defined in Section *Domains of influence and occupations*. The following log transformation has been applied to the time axis: $year\to 7.6-log(1991-year)$. The “Other” category is not displayed in the bottom chart due to its limited size.

Figure 3 reports the time evolution of the share of female individuals. It exhibits a U-shape pattern with a local minimum at around 5–10% of female individuals around 1750. At the end of the observation period, the share of female individuals ranges between 20% (Africa) and 30% (Asia and Oceania). The main reason for this rising share in the database is the growing influence of two categories, Sports and Culture, that are much more balanced across genders than categories such as Governance/Executive and Academics. If the share of women is higher in the English edition over the entire sample period (see statistics in Table S4), the Western non-English edition further mitigates the bias after 1950, with a sample average close to 27% of women, as opposed to a 22.5% in the English edition.

#### Politics and the emergence of modern democracy

**Fig. 4:**
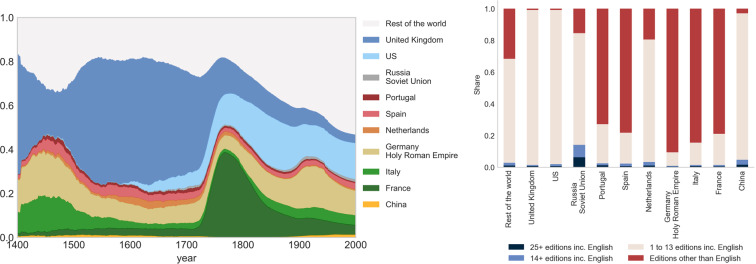
Evolution of the share of individuals per citizenship identified as “politics”, 1400–2000AD. Notes. Cross-verified, restricted sample (at least one Wikipedia edition among the 7 European languages analyzed), see Section *Extracting biographic information from a restricted sample*. The occupations are defined in Section *Domains of influence and occupations*. For a given year, the number of living individuals is calculated by summing up all individuals such that $birth\_date\le year\le death\_date$. When not available, the date of birth (resp. death) is estimated from the estimated average longevity over the period. Left panel: Most popular citizenships (share of living individuals on the vertical axis, year on the horizontal axis). Right panel: share of individuals with biographies in a) 25+ editions including English, b) 14+ editions including English, c) 1 to 13 editions including English, d) One or more editions than English), breakdown by citizenship.

Figure 4 documents the number of individuals associated with politics. This illustrates the rise of American politics in the second half of the 18th century at the time with the birth of the independent nation of the USA, as shown in the left panel of Fig. 4. The same holds true for France: at that time, the share of French and US politicians in our database is rising in a significant way around and after the end of the 18th century. While the emergence of modern democracy in those countries is noticeable on this graph, the main feature here is the dark blue part in the 16th century in British Islands, which saw the gradual integration of the four kingdoms of England, Wales, Scotland and Ireland. Many historians consider that central elements of modernity, of capitalism, industry, and science, originated in early modern Britain (circa 1500–1750)^15,16^. In the right panel, we display the relative importance of different language editions other than English - see e.g. the importance of red bars featuring individuals not present in the English edition of Wikipedia. This is particularly large for French individuals (80% individuals), and true for other citizenships including Italians (+70%), Germans (+85%), Portuguese (+70%) and Spanish (+65%) individuals. For this specific occupation (politics), restricting the sample to English pages would drastically change the relative importance of citizenships, for instance almost entirely removing French politicians and considerably expanding English and US ones.

#### The age of exploration and discoveries

**Fig. 5:**
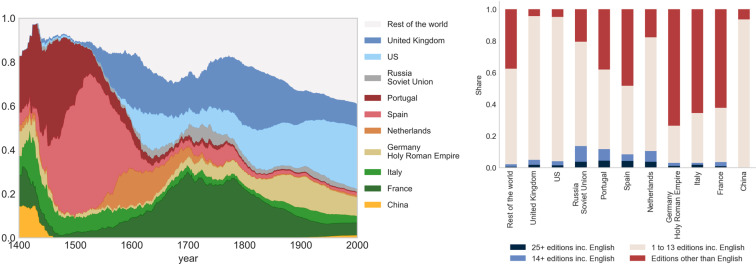
Evolution of the number of share of individuals per citizenship identified as “explorer, inventor, developer”, 1400–2000AD. Notes. Cross-verified, restricted sample (at least one Wikipedia edition among the 7 European languages analyzed), see Section *Extracting biographic information from a restricted sample*. The occupations are defined in Section *Domains of influence and occupations*. For a given year, the number of living individuals is calculated by summing up all individuals such that $birth\_date\le year\le death\_date$. When not available, the date of birth (resp. death) is estimated from the estimated average longevity over the period. Left panel: Most popular citizenships (share of living individuals on the vertical axis, year on the horizontal axis). Right panel: share of individuals with biographies in a) 25+ editions including English, b) 14+ editions including English, c) 1 to 13 editions including English, d) One or more editions than English), breakdown by citizenship.

Figure 5 (left panel) shows the share of individuals in the *Explorer Inventor Developer* category by citizenship. It illustrates the end of the chinese exploration period which lasted until the 15th century followed by the European age of discovery and explorations conducted by Portugal and Spain in the 15th and 16th centuries.

These two European waves of exploration are imperfectly covered by the English edition. In the right panel, the fraction of these individuals (red bar) not included in the reference edition ranges between 10% and 15% for Portugal and Spain, respectively. The Chinese exploration benefits from a better coverage in the English edition. The contribution of the present project is even more striking when one considers the share of individuals not present in existing projects (pink and red bars). At the margin, we add almost 75% and 80% of the Portuguese and Spanish individuals of the category here. The dark green peak on the graph corresponds to the French Renaissance period (between the 15th and early 17th centuries). This important period of human history is not covered in a satisfactory way in the English edition again and therefore in existing projects. The share of individuals retrieved with the French citizenship in editions other than English is at around 25% (red bar). If we add to this the fraction of individuals ignored so far in existing databases (65%) we can conclude that we improve coverage by almost 90%. A similar pattern is observed for Italians earlier during the Italian renaissance period which spans from the end of the 14th century (Trecento) to the early 16th century (Cinquecento). Indeed, in the figure, a thick band of light green is visible over the entire period. It becomes thinner thereafter. The emergence of the US is also noticeable over the 20th century, when many inventors and creative people of all kinds were attracted thanks to a favorable immigration system aligned with a dynamic academic sector. We indeed observe this increasing share (light blue) of the US during that period in the category. These individuals all have a biography in English, but were missed by more selective databases: around 90% or more of these individuals were present in less than 14 Wikipedia editions.

#### Conjecture: the role of science and politics in escaping the Malthusian trap

**Fig. 6:**
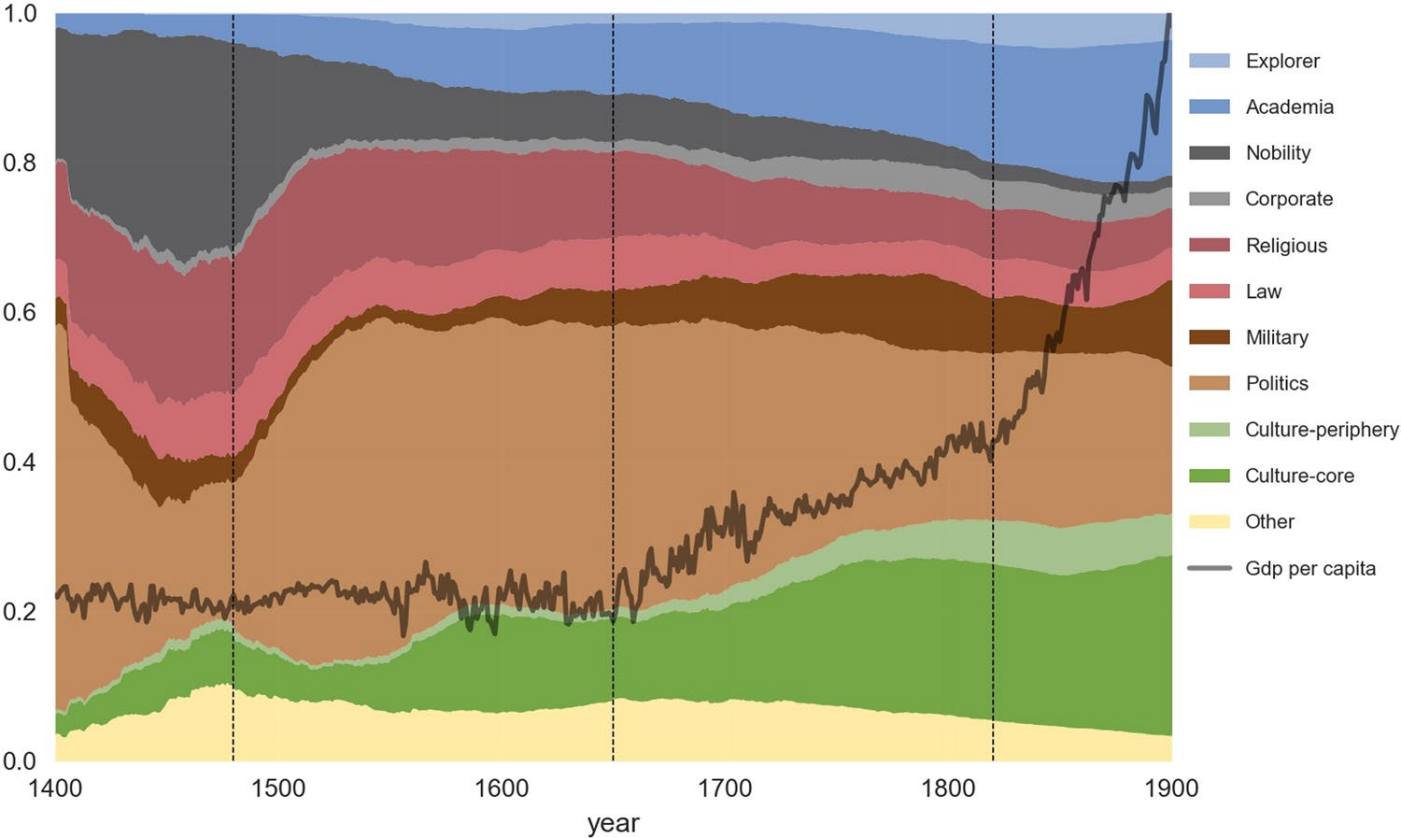
Share of individuals in the database, breakdown by domain of influence, and GDP per capita, Country = United Kingdom. Notes. Cross-verified, restricted sample (at least one Wikipedia edition among the 7 European languages analyzed), see Section *Extracting biographic information from a restricted sample*. The vertical axis represents the share of individuals per occupation and GDP per capita (source Maddison database) normalized to 1 in year 1900. Occupations are defined in Section *Domains of influence and occupations*. For a given year, the number of living individuals is calculated by summing up all individuals such that $birth\_date\le year\le death\_date$. When not available, the date of birth (resp. death) is estimated from the estimated average longevity over the period. The vertical bars in 1650 and 1820 correspond to the two changes in the GDP per capita, the second one being associated with the Industrial Revolution. The vertical line in 1480 corresponds to the increase in the number of notable individuals classified in Politics and the decrease in the corresponding Nobility category. Restricted sample (at least one Wikipedia edition among the 7 European languages analyzed). Comparison with GDP per capita. Imputed life time when missing.

Theories of long run economic growth^17,18^ explain that the main reasons for escaping the Malthusian trap (where improvement due to technical progress allows population increases but no change in income per capita) are due to the accumulation of *latent* factors such as human capital and the evolution of political regimes around 1750AD in Western countries. A first insight could be derived from Fig. 2 where, in the two centuries before that year, one observes a rise of Academia and Politics, and a decline in Religion and Nobility. In Fig. 6, we take a closer look at the UK, the country leading the Industrial Revolution. The figure shows two distinct regimes of GDP per capita growth after the initial Malthusian stagnation: a first economic expansion in the late 17th and 18th century, that follows a century of a latent relative expansion of “Politics” with Nobility declining instead; a second regime of the Industrial Revolution of the early 19th century, which is preceded by the latent relative expansion of science and the arts. An overlook at other countries (unreported) suggests that similar patterns emerge with a particularly strong role of the composition of the leadership category which contains Nobility, Religion, Military and Politics. Previous economic history work^19^ offers a detailed review of the arguments in favor of the emergence of new technologies and the respective role of Enlightment vs. trade and profitability argument to explain the economic take-offs.

#### Geolocalisation

**Fig. 7:**
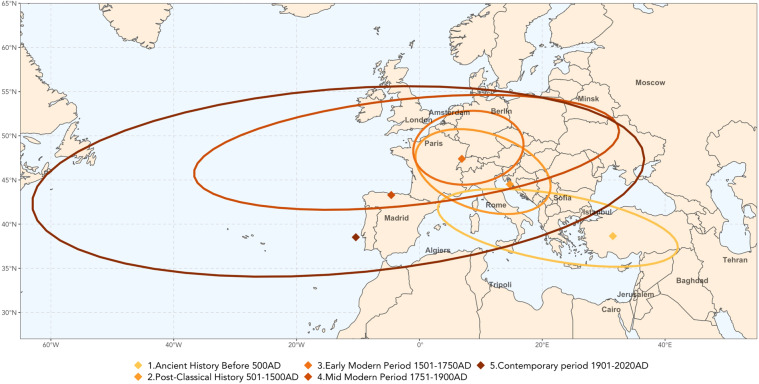
Barycenters and ellipses of covariance matrix of longitude and latitude of birthplaces per period of human history. Notes. Cross-verified, restricted sample (at least one Wikipedia edition among the 7 European languages analyzed), see Section *Extracting biographic information from a restricted sample*, and a known birthplace. The barycenter is computed as the arithmetic mean of the longitudes and latitudes of the birthplaces. The function stat_ellipse of the package ggplot2 of R is used to draw Gaussian ellipses in which 40% of the population is concentrated.

**Fig. 8:**
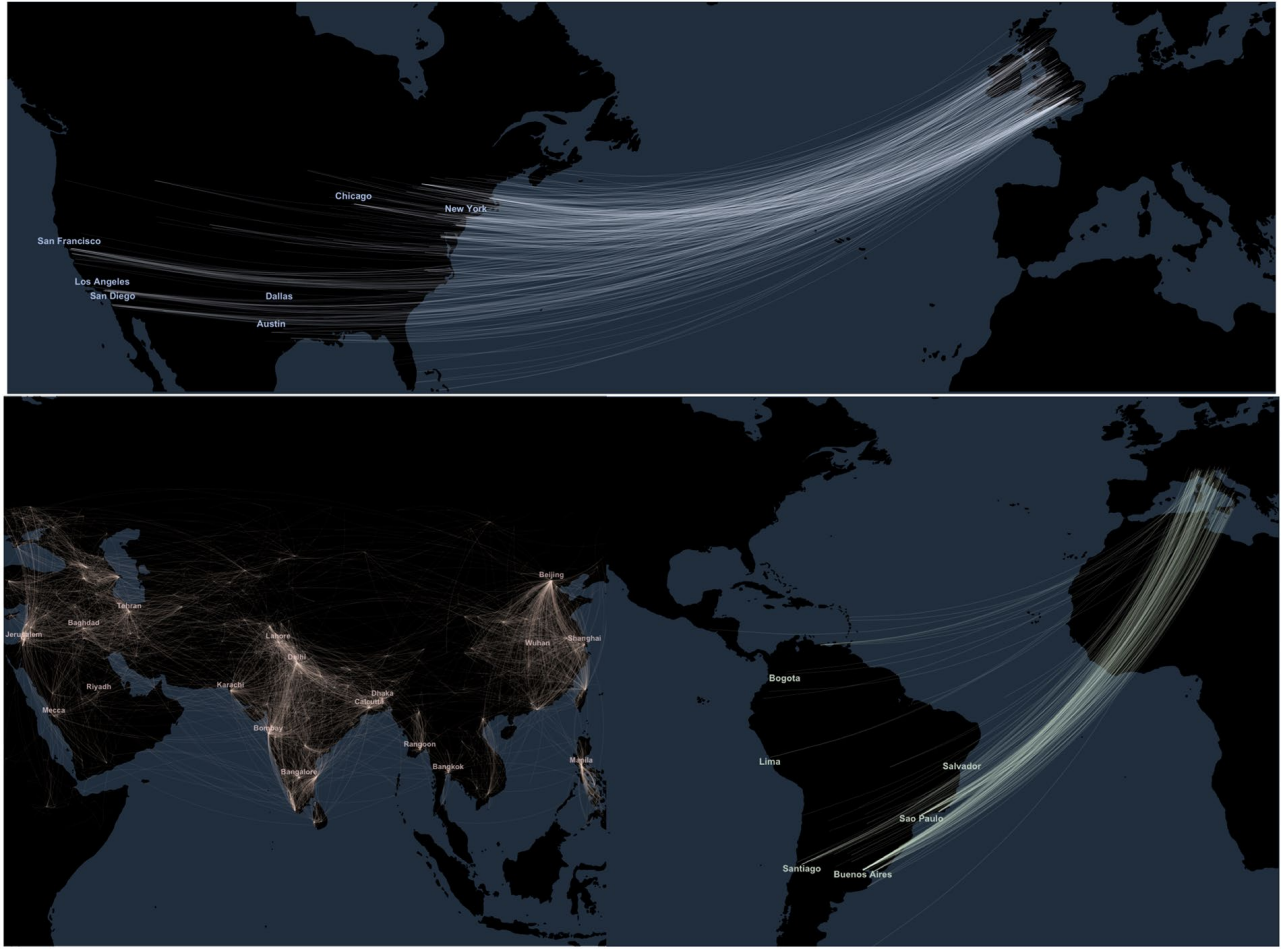
Migration charts, selected countries and domains of influence. Notes. Cross-verified, restricted sample (at least one Wikipedia edition among the 7 European languages analyzed), see Section *Extracting biographic information from a restricted sample*, and both a known birthplace and deathplace. Top chart: birth to death flows of academics from the UK to North America. Bottom chart: on the right, birth to death flows of notable individuals in culture from Italy to South America; on the left, birth to death flows (all categories) within a rectangular area encompassing Asia including Central Asia, Western Asia and the southern part of Eurasia. All curves connect the city of birth and of death of individuals.

Using longitudes and latitudes of birth and death places, one can track the evolution of the global geographical centers of the database. Fig. 7 represents the ellipse of the variance-covariance matrix of longitudes and latitudes of birthplaces at different periods of time (40% of the sample population lies within each ellipse). Fig. 8 represents the birth to death flows and generalizes early pioneering works^1,2^. The higher granularity of our database allows us to cover less explored destinations or smaller geographical divisions. Going from the 150,000 individuals in earlier studies to 2.3 million individuals in our case allows us to cover migration outflows of, e.g. artists who left Italy to emigrate in numbers to South America, or scientists who left the UK to live in North America. We can also represent intra-Asian migration flows which is mostly within countries contrary to inter-country European flows.

#### Outreach

Our database can be used to provide a new level of granularity and historical depth to several fields. In urban economics, one may test the quantitative implications of the model of cities attracting talents^20^, the role of the creative class to enlighten the causation chains^4,21,22^, the impact of transport infrastructure development on geographic mobility^23,24^, or the existence of gravity forces in trade to uncover ancient cities as trade places^25^. Many gender issues can be revisited with greater historical depth and finer geographical scale, like the importance of role models in the dynamics of women’s empowerment thanks to alternative measures of notability and the use of geo-coded birthplaces^26,27^, the importance of the local environment enabling women to become inventors^28,29^, the convergence of male and female occupations over time^30^. The database can similarly be used to study several long-run issues. For instance, to the extent that the attractiveness of cities for scientists and artists are a proxy for economic growth and the notable people in law, governance and administration capture the quality (either positively or negatively) of institutions, the database can be used to investigate the role of institutions on growth^15,31,32^. It offers testable implications of the unified growth theory, such as the replacement of physical by human capital^17,18^, as well as neo-Schumpeterian theories and the role of innovation and competition^33,34^, thanks to our account of scientists and innovators per country. The same is true for detecting evidence of cultural transmission mechanisms through naming^35–37^, or to deepen our understanding of the causes and consequences of key historical events such as the Protestant reformation along the lines of pioneering works^38–40^ as well as the role and determinants of culture in the wealth of nations^41–43^. Research in demography economics for instance on longevity in space and time^5^ can be enriched thanks to our list of birth and death dates by field. Issues on international and domestic migration can also be revisited from a more longitudinal perspective. For example, Zelinksy’s theory of mobility transition^44^, the theory and evidence on brain drain^45^, the birthplace diversity and economic prosperity^46^ and the relationship between future outcomes and birthplace^47^ can be revisited from a historical perspective, thanks to the geocoded birth and death locations and occupation variables. Domestic migration within the United States and immigration to the United States and its links to economic prosperity^48^ or extra-place based mortality^49^ can be revisited with the use of geocoded birth and death locations in our dataset, with but possibly even without matches with the historical US censuses. Beyond these economic approaches, and without exhaustivity, political scientists can use the database to analyze the evolution of political ideas and the way they interact with the development of democracy; given the mass of data, historians may identify more easily new key protagonists of most historical periods; similarly, sociologists may identify individuals who played or are still playing a role in subculture; demographers could use the demographic information to compare life expectancy of the subgroup of notable individuals in different regions or to the general population.

## Data Records

The cross-verified dataset is available at the publicly accessible Sciences-Po Dataverse: 10.21410/7E4/RDAG3O^50^. Each entry is characterized by the following set of variables:

- *name*: full name of the individual;
- *group_wikipedia_editions*: partition category of the individual (from A (\textit{English} edition of \texttt{Wikipedia}) to F (\texttt{Wikidata} only) as described in Table 1.);
- *birth*: birth date of the individual (either reported or estimated);
- *death*: death date of the individual (either reported or estimated);
- *level1_main_occ & level2_main_occ & level2_second_occ & level3_all_occ & level3_main_occ & freq_main_occ & freq_second_occ*: set of seven variables for the main domain of influence of each individual in three layers (level 1: 6 groups; level 2: 15 sub-groups and the frequency we impute to the second domain if multiple domains); level 3: keywords collected to assign a domain;
- *gender*: gender of the individual;
- *area_of_rattachment 1 and 2*: first and if needed, second citizenship of the individual with a reference to the current country or to a former political regime;
- *ranking_visib_5criteria*: computed from 5 variables: number of Wikipedia editions, non-missing biographic information, length of pages, hits of pages and total number of external links. An alternative ranking based on the sum of the log of these variables plus one is in *sum_visib_ln_5criteria*;
  - *number_wiki_editions*: number of different Wikipedia editions;
  - *non_missing_score*: total number of non-missing items retrieved from Wikipedia or Wikidata for birth date, gender and domain of influence;
  - *total_count_words*: total number of words in all biographies from Wikipedia;
  - *wiki_readers_2015_2018*: average per year number of page views in all Wikipedia editions (information retrieved in 2015–2018);
  - *total_noccur_links*: total number of external links (sources, references, etc.) from Wikidata;
- *bplo1 & dplo1 & bpla1 & dpla1 & birthplace_name & deathplace_name*: longitude, latitude and name of birthplace, deathplace. To be used with caution, the accuracy of these variables has not yet been verified.
  The exhaustive dataset is available at the publicly accessible Sciences-Po Dataverse: 10.21410/7E4/YLG6YR^51^ This dataset is not cross-verified and should not be used directly or under the full responsibility of users. It includes all the variables detailed above. The following five variables:
  - *exhaustive_level1_main_occ & exhaustive_level2_main_occ & exhaustive_level2_second_occ & exhaustive_area1_of_rattachment & exhaustive_area2_of_rattachment*
    correspond to the variables
  - *level1_main_occ & level2_main_occ & level2_second_occ & area1_of_rattachment & area2_of_rattachment* in the restricted dataset.

## Technical Validation

This section provides some discussion concerning the bias of our restricted sample detailed above: notable individuals with at least one biography among the universe of Wikipedia editions we considered (7 European language editions) plus a Wikidata entry.

### Missing information

Table 3 documents the share of missing information of the main variables, depending on the language group: English versus Western non-English. The first column contains the proportion of individuals with no birth date information (exact or approximate). The proportion is quite low for the Western non-English group (around 2%) while this rate becomes four times larger for individuals in the English group (around 8%). The proportion of missing death date is not included in this table as around 50% of notable individuals are still alive. In Columns (2) to (4), the proportion of individuals with no gender, occupation, and citizenship is very low in both samples. These low rates of missing information result from our initial choice to work on a restricted sample of individuals with at least one biography in Wikipedia. People present in both groups (English and Western non-English) are the most famous ones and very few information are missing for these individuals. In column (5), the proportion of individuals with no birthplace is quite high in both samples (34% for the English group and 29% for the Western non-English group). Finally, the last column contains the proportion of individuals for whom no information is missing. In both groups, the rate is quite high with 64% in the English group while it is at around 69% in the Western non-English group.

**Table 3:** Freq. missing information vs. complete profile.

*Wikipedia* (by. language group)	Birth %	Gender %	Occupation %	Citizenship %	Birthplace %	vs. Complete profile %
English	8.15	0.09	0.42	2.31	34.25	64.07
Non-English	2.27	0	1.2	2.38	28.87	69.39

Notes. Restricted sample (at least one Wikipedia edition among the 7 European languages analyzed), see Section *Extracting biographic information from a restricted sample*. The occupations are defined in Section *Domains of influence and occupations*. First 5 columns: % missing observations; last column: % with no missing observation among these 5 variables.

### Comparison between Wikipedia editions and Wikidata

The strength of our methodology comes from the fact that we use 7 editions of Wikipedia and Wikidata in order to cross-verify the maximum number of information. Although the convergence rate is quite high, it happens in rare occasions that Wikipedia editions and Wikidata contradict each other on some dimensions. Table 4 reports the number of individuals for whom one information (e.g. birth year) has been detected in both sources (row #1) and its share in the sample (row #2). The third and fourth rows give the discrepancy rate between Wikipedia and Wikidata on each dimension. Concerning dates, the share of exact mismatch is very low (row #3): 2% (birth) and 1.6% (death). Larger differences (10+ year difference) are even less frequently observed (row #4): 0.23% (birth) and 0.38% (death). The discrepancy rate for gender is also very low at around 0.51%.

**Table 4:** Wikipedia & Wikidata: similarity and differences.

	Birth	Death	Gender	Occupation	Citizenship
P and D coincide (# Obs)	2,011,546	921,231	2,239,532	1,941,246	1,748,981
P and D coincide (%)	87.77	40.20	97.72	84.70	76.31
P and D differ (conservative %)	2.14	1.64	0.51	11.04	9.93
P and D differ (flexible %)	0.23	0.38	n.a. (*)	7.17	n.a. (*)
D (missing) and P (available) (# Obs)	62,263	30,070	15,372	186,951	393,198
**D (missing) and P (available) (%)**	**2.72**	**1.31**	**0.67**	**8.16**	**17.16**
Mismatch (and source = P) (# Obs)	1,032	484	0	9,528	19,591
Mismatch (and source = P) (%)	0.05	0.02	0.00	0.42	0.85

Notes. Restricted sample (at least one Wikipedia edition among the 7 European languages analyzed), see Section *Extracting biographic information from a restricted sample*. The occupations are defined in Section *Domains of influence and occupations*. This table provides some summary statistics on similarity and differences between the sources Wikipedia (P) and Wikidata (D). P and D differ (flexible %) difference in terms of birth/death years between P & D lower than 10 years (for columns Birth and Death), or difference between large categories between P & D (column Occupation). Conservative means that an exact match is required (for years of birth and death or for level 2 occupations). (*): n.a. = distinction not applicable.

The information on the domain of influence is not consistent in a larger number of cases (11%). Most of the time this is due to different sub-categories detected that are usually close to each other, e.g. Culture-core vs. Culture-periphery or Politics vs Military, or Nobility vs Family. To get rid of such minor differences we compute similar discrepancy rates considering the following set a broader categories: Discovery/Science, Culture, Leadership, Sports/Games, Other. As expected, the discrepancy rate shrinks to 7.2% (row #4) for occupations. Mean discrepancy rates for citizenships are at around 10% (last column). Such high rates mostly reflect the fact that Wikidata reports the regime in force at the time when the individual lived while Wikipedia reports more often the current regime information. For instance, Martin Luther’s country of citizenship is “Electorate of Saxony” (which belongs to the Holy Roman Empire) in his Wikidata biography (https://www.wikidata.org/wiki/Q9554) while his English Wikipedia biography (https://en.wikipedia.org/wiki/Martin_Luther) says he “was a German professor of theology”.

Subsequent rows (#5 and #6) illustrate the importance of considering different sources: they show how frequently one information is found in Wikipedia while absent from Wikidata. These statistics are useful to figure out the marginal contribution of Wikipedia which is sizeable. Indeed, combining Wikipedia and Wikidata adds a substantial fraction of observations to the database. For instance, Wikipedia brings in 2.72% additional birth dates, 1.31% additional death dates, 0.67% additional gender information, 8.16% additional occupations and 17.16% additional citizenships. While the contribution of Wikipedia appears limited on birth (+2.72%) and death dates (+1.31%) as such basic information are most often reported in Wikidata, Wikipedia proves much more useful to complement Wikidata on occupations (+8.16%) and citizenships (+17.16%). The last two rows report the number of cases with discrepancy between Wikipedia & Wikidata and for which we selected Wikipedia, for a small proportion of cases.

### Manual checks

We ran several tests to check the accuracy of our dataset. We hired three teams of students in three areas (from France, the UAE and India) representing very diverse cultures to compare the information contained in our final database with the information present in Wikipedia/Wikidata. We asked them to report mistakes on 6 different dimensions (exact or approximate birth and death dates, gender, main occupation and possibly secondary occupation, citizenship and possibly secondary citizenship). The 5 different possible outcomes are as follows: 1) Information not reported in our database: a) “No info. in sources” means the information is not included in Wikipedia/Wikidata nor in the dataset, b) “Info. updated since data collection” means the information is included in the current version of Wikipedia/Wikidata but was not present in this universe at the time of collection; 2) Information reported in our database: a) “Correct” means no error, b) “Error” means certain error, c) “Other case” means possible error (for instance historical controversy, several sources contradicting each other or information updated since data collection).

A pilot wave on 5 sub-samples of 500 individuals each, with a 20% overlap, led to a percentage of errors below 1% except for the first and second occupation, that is above 1%. The final test is based on a larger sample: 10 students received a sub-sample of 1000 individuals each, with exact overlap so that every individual would be checked twice. The total sample of 5,000 individuals thus verified contains the top 1,000 individuals of the database, 500 individuals with two biographies in Wikipedia at least (these are all present in the top quartile of the visibility distribution and the median individual in that sample was in the 92th centile of the visibility distribution), 3,000 individuals from the database with the 4 quartiles equally represented, and finally 500 individuals with no Wikipedia biography, and only a Wikidata entry. See Appendix C for more details on the instructions given to the RAs. In addition, one individual was dropped, because it had been selected twice. To conclude, we manually verified the information for 4,999 individuals.

An information is deemed valid when two independent research assistants assessed its correctness. For cases where their assessment diverged, we asked a PhD researcher to double check the information and try to determine the origin of that discrepancy. See Table S5 in Appendix C where we report the various discrepancy rates for each variable.

**Fig. 9:**
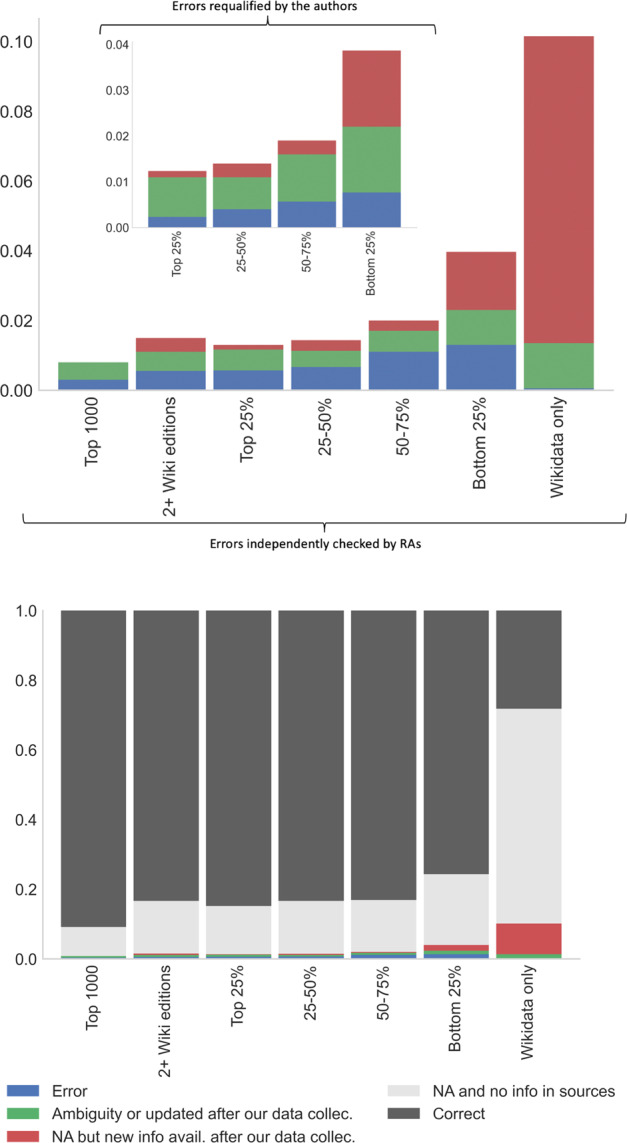
Manual verifications: summary statistics. Notes. Top panel, larger graph: the statistics verified independently twice by a team of 10 RAs and cross-verified by a PhD researcher. Focus on errors, imprecisions or updates since our data collection. Smaller graph on top: requalification of some of the errors by the authors. Vertical axis represents the percentage of errors divided by the number of observations including NA (themselves approx. 20% of total number of observations). The level 0.005 on these graphs represents 0.5% of all observations and 0.5/(1-0.2) = 0.625% of all non-missing observations. Bottom panel: correct information, missing, errors, imprecisions or updates since our data collection, verified independently twice by a team of 10 RAs and cross-verified by a PhD researcher. See also Appendix C (Tables S6 to S10) which provides the summary statistics concerning manual checks for each of the following variables: birth date, death date, gender, first and second occupations, first and second citizenships, for all sub-samples.

Our verification allows for a clear distinction between the errors due to the algorithm and the errors due to the evolving nature of Wikipedia. We report in Fig. 9 the distribution of various cases for the different samples (top panel). The error rate is increasing for individuals with shorter biographies, as expected, going from less than 0.3% for the top 1000 individuals and 0.5% for the most documented individuals in the top quartile to almost 1% in the bottom of the distribution. There is also a large number of missing information in the Wikidata only sample.

The authors finally verified the errors detected independently by the different teams of RAs and the PhD researcher, and requalified some of them after a final cross-examination. Most of the errors detected by the RAs were actually due to a slight discrepancy - typically one year - between the birth date contained in the database on one side and what was found in our reference sources. Most of the time Wikipedia and Wikidata were themselves conflicting on these cases (e.g two biographies would report respectively 1944 and 1943 and we had reported 1943). So the numbers reported tend to overestimate the true number of errors by a factor 3 to 6. We display the number of errors after we requalified them in the smaller chart in the top panel. We also report in green the fraction of cases where the information was not available in our database but became available after our data collection. The bottom panel reports the shares of correct information and the share of cases where the information was not available in our database and was still missing in both reference sources.

### Western bias

In this paper, we introduced a multi-language database of notable individuals with the use of 7 language editions of Wikipedia and Wikidata to assemble a list of 4,678,040 individuals. This significantly reduced the Anglo-Saxon bias, but not all. Two main drawbacks remain. First, we did not exploit the non-Western language editions to cross-verify information on individual characteristics. Second, we did not collect the number of words beyond these 7 language editions: they enter in the notability index, but this index cannot be considered as global, resulting in a Western-world bias in notability measures. This is however partly compensated by the use of the total number of hits for all Wikipedia editions and not only 7, in our aggregate notability measure.

### Errors in Wikipedia

The accuracy of Wikipedia being not perfect^7,8^, our data is as good as the source data, but our approach adds new possibilities: to cross-check across different language editions and reduce errors when possible.

## Usage Notes

The full data set will be on our dedicated website here https://medialab.github.io/bhht-datascape. The data are available both in.csv and Stata (.dta) format. The restricted database was cross-verified. It is subject to CC-BY-SA licensing. The intermediate files as well as the exhaustive database are not cross-verified and should not be used directly or under the full responsibility of users.

## Supplementary information

Online Appendix: supplementary tables and figures

## Data Availability

Code to replicate all analyses presented here, the intermediate datasets and the exhaustive dataset are available at the publicly accessible Sciences-Po Dataverse: 10.21410/7E4/YLG6YR^51,52^.
