# Supplementary material for: A cross-verified database of notable people, 3500BC-2018AD
Source: Sci Data. 2022 Jun 9;9:290. doi: 10.1038/s41597-022-01369-4 (PMC9184645; doi:10.1038/s41597-022-01369-4)
Supplement: Supplementary file 1 — Online Appendix: supplementary tables and figures [file 41597_2022_1369_MOESM1_ESM.pdf]

Online Appendix: supplementary tables and figures

A Data collection details and processing for the exhaustive sample

A.1 Selection into **Wikipedia**

The selection rules for Wikipedia entries are category-specific. They are in particular specific for living persons, and described here: https://en.wikipedia.org/wiki/Wikipedia:Biographies_of_living_persons, as well as general guidelines for notability: https://en.wikipedia.org/wiki/Wikipedia:Notability_(people).

Rules differ marginally across language editions; rules are specific to the type of human activities. It is beyond the scope of this paper to systematically discuss these rules but a few principles emerge: i) one should avoid biographies based on a unique, arbitrary source (see subsection B.3), as in the universe of Wikidata only (no biography in Wikipedia), which adds millions of individuals from unverified sources and include homonyms and duplicates; ii) biographies of *living* persons should be used with caution and stricter criteria such as the existence in several language editions should be applied; iii) some categories are more likely to be subject to idiosyncrasies (judgment call) of contributors, in particular those related to family members, criminals, victims of accidents, athletes with no international recognition. These considerations motivate our restrictions on the sample studied throughout this paper.

A list of urls of individuals who died in 1953 can be found here: https://en.Wikipedia.org/wiki/Category:1953_deaths. The corresponding urls in the French (resp. Portuguese, Spanish, Italian, German and Swedish) edition were accessed by using “fr” (resp. “pt”, “es”, “it”, “de”, “sv”) instead of “en” in all urls. In the particular case of France, Wikipedia sorted individuals by month-year of birth and death, so the loop for scraping individual biographies was adjusted accordingly to cope with this monthly frequency.

A last issue affecting selection is the so-called *survival bias*, which states that we only observe the characteristics of the survivors and those could be biased - those present in the dataset but not those who may have had an impact - they would be in the set we called $\mathfrak{F}$. We can, however, approximate the rate at which people survived to make it into the final dataset, under the assumption that the fraction of notable people affecting society at the time they lived is a constant of the living population at that time, and that they are forgotten at a constant rate per unit of time. This is of course a pure thought experiment but provides an order of magnitude of the number of notable individuals we may still be missing.

A.2 Removing duplicates: details

Dealing with possible duplicates is not an easy task as we need to separate these cases from real homonyms, i.e. individuals sharing exactly the same name and first name. We use a total of eleven methods, all detailed below, ranging from string normalization, phonetic encoding and string distance metrics to identify likely duplicate pairs that we eventually decide to merge by manually checking their respective Wikipedia biographies. In order to reduce the number of candidates, which is prohibitively large in our database, we determine a score for each candidate based on some additional features such as common birth or death dates, the citizenship and domains of influence retrieved from these questionable biographies. This helps us discard candidate pairs which were not duplicates.

We then construct a score ranging from 0 to 1 which corresponds to the likelihood for a set of biographies to correspond to the same individual. A score above 0.75 for 4 criteria and above 8 for the remaining two identifies a ’cluster’ of individuals who have a high probability of being the same person; and we kept the person with the highest number of available biographical information. We identify 34,562 true duplicates, that is 0.7% of the total number of individuals (34,562/4,678,040).

We use the following methods to remove duplicates:

1. Connected components solving: sometimes links between Wikipedia biographies are not mutual. It is therefore possible, by gathering connected components of the page lowercase names’ graph, to find suitable duplicate pairs.
2. Aggressive string normalization: by normalizing hyphens, underscores, solving url encoding and dropping non-alphanumeric characters, one can find more suitable duplicate pairs.
3. Unicode standardization: some languages, such as English, do not handle accentuated characters very well and tend to avoid using them. By standardizing unicode characters to plain ascii, it is possible to match similar names in two different languages. It is also possible to match names written in other alphabets thusly.
4. String fingerprinting: there is a large variety of ways to write the same name. It is not rare, for instance, to see Asian names written in the incorrect order by occidental clerks. String fingerprinting is a method which applies a set of transformations to a string to normalize order, redudancy and case so one can match similar-looking strings.

5. Squeezed string fingerprinting: same as before except that we will "squeeze" consecutive duplicate letters into a single one. For instance, the name "Brettner" would become "Bretner". This follows the observation that double letters tend not to be well-respected across variants of the same name.
6. Small tokens filtering: small tokens composed of only one or two characters, such as "de" or "of", and stopwords tend to be frequently forgotten in names. Filtering them will produce some more matches.
7. Rusalka phonetic encoding: by producing a symbolic phonetic representation of the considered names, one is often able to match different transliterations or spellings.
8. Sorted neighborhood using the omission key and Levenshtein distance less than or equal to one: string distances such as the Levenshtein distance are very useful to find similar-looking strings. Unfortunately, a naive approach to collect pairs of duplicates in a dataset results in quadratic processing time. While this is acceptable for tiny datasets, it is not for millions of names. The sorted neighborhood method can approximate pairwise computations by considering that if you order strings using a specific key beforehand then similar pairs have a high probability of being close in the sorted list. A fixed-size window is then slided across the sorted list where pairwise distances are computed and similar pairs reported. We first choose to use the omission key, a string's key leveraging the frequency to which characters are omitted when misspelling words, to sort our dataset before proceeding to find pairs having a very low Levenshtein distance.
9. Sorted neighborhood using the skeleton key and Levenshtein distance less than or equal to one: same as before but using a different key, the skeleton key, leveraging the way words tend to be misspelled in the English language, i.e. misspelled consonants are frequently not the first ones.
10. Cologne phonetic encoding: this phonetic encoding targets specifically German and similar languages and is a good complement to the Rusalka one. Its precision is very low however since it tends to approximate sounds a lot.
11. Sorted neighborhood using the skeleton key and Levenshtein distance less than or equal to two.

    Further references[1–4].

A.3 Data collection using categories

We develop a methodology based on the information found in the categories of `Wikipedia` to approach the universe of notable individuals. We scraped individuals from a particular procedure based on categories. Categories are present in the bottom part of most biographies. These independent `Wikipedia` objects contain lists of individuals (and their associated urls) who have one feature in common such as such as: birth date, death date, domain of influence, etc. In a first stage, we harvest all links available in the "Living People" (https://en.Wikipedia.org/wiki/Category:Living_people) category of the English edition. In a second stage, we explore additional categories such as "Possibly living people", "Deaths (resp. birth) by year", "Deaths (resp. birth) by decades", "Deaths (resp. birth) by centuries" and "Deaths (resp. birth) by millennium", etc. to collect more urls. Last, we parse the following list of categories to detect individuals that were not identified in the previous stages: "Date of birth missing", "Date of birth unknown", "Date of death missing", "Date of death unknown", "Year of birth missing", "Year of birth unknown", "Year of death missing", "Year of death unknown", "Place of birth missing", "Place of birth unknown", "Place of death missing", "Place of death unknown".

A.4 Oldest registered entries and comparison with world population estimates

The first registered human in our database was born around 430,000BC, namely "Cranium 17", an ancient hominid skull. The second oldest entry, 11,000BC, is a skull of a Paleo-Indian woman discovered in Mexico city in 1959. Three other famous skeletons (the Kolebjerg Man (8000BC), Loschbur-Fra (8000BC), the Frau von Bäckaskog (7000BC)) follow. The first individual with a social status comes next in 6th position in our database. Pesho, was "*chief, who lived ca. 7000-7500 years ago in territory of modern Bulgaria and known for his rich tomb (sic).*" The first notable individual, in the sense of his prominent role in history, comes next in 7th position. Ny-Hor, born between 4000BC and 3001BC, was *"a king in the Egyptian predynastic period, and known as Her or Hor (Horus), that is, "the Falcon", and his monarchy is established in Nekhen (later Hieraconpolis).*" Interestingly, this Pharaoh has a biography in French, Arabic, German, Russian, Italian, Portuguese and a few other languages but, as of June 2019, not in English. We arbitrarily decided to officially start our database with this political figure. Later on, there would be more famous individuals such as the king of Tyre Deleastartus and the Assyrian kings Puzur-Ashur I and III (circa 2000BC and 1500BC respectively), the Chinese empire chancellor Yi Yin (born 1648BC) and the sixth king of Babylon Hammurabi who died in 1750BC. In total, our database contains 330 individuals who have lived before Hammurabi, the sixth king of Babylon, who is the oldest registered notable individual in earlier sources[5].

One can compare the evolution of the living notable persons in our database to world population and world GDP. We use two sources for population[6,7], which are extremely close to each other over the most recent period we consider (1000BC to now). World GDP estimates also comes from the first source[6]. The series are represented in Figure S1, in log, and the x-axis is either linear or a log of the calendar year. All series grow, but the notable population in the database drops before 500BC; the world population increases fast in the last two centuries, but the population of notable people increases faster and is more in line with GDP growth.

Table S1. Oldest individuals in the exhaustive database

Name	Wikidata Code	Birth Min	Birth Max	Death Min	Death Max
Cranium 17	Q41330363	-430000	-429001	-430000	-429001
Femme de Peñon	Q1988410	-11000	-10001	-11000	-10001
Koelbjerg Man	Q455750	-8000	-7001	-8000	-7001
Loschbur-Fra	Q25583326	-8000	-7001	-8000	-7001
Frau von Bäckaskog	Q6981339	-7000	-6001	-7000	-6001
Pesho	Q29510353	-5000	-4001	-5000	-4001
Ny-Hor	Q268647	-4000	-3001	-4000	-3001
Mummia del Similaun	Q171291	-3345	-3345	-3255	-3255
Menes	Q189574	-3200	-3200	-3100	-3100
Hat-Hor	Q577451	-3150	-3150	-3095	-3095
Frau von Luttra	Q179281	-3125	-3125	-3100	-3100
Djer	Q152375	-3000	-2901	-3000	-2901
Djet	Q151828	-3000	-2901	-3000	-2901
Merneith	Q230548	-3000	-2901	-3000	-2901
Teti I.	Q153154	-3000	-2901	-3000	-2901
Den (pharaoh)	Q151822	-3000	-2901	-2995	-2995
Semerkhet	Q151805	-3000	-2901	-2960	-2960
Nefer (Hofzwerg)	Q1800518	-3000	-2901	-2900	-2801
Iblul-II	Q4202987	-3000	-2001	-3000	-2001
Mann von Porsmose	Q1726357	-3000	-2001	-3000	-2001

Notes. Exhaustive sample (4.7 million individuals). The birth and death min and max are based on the precision of the related dates: millenia, centuries.

Figure S2 provide the split over 4 sub-periods of the ratio of world population to the population of notable individuals. Our database population contains approximately one person out of 250 000 before 500AD, the ratio then declines continuously until one out of 50 000 in 1500AD, continues declining to reach a local maximum in 1700 due to a larger mortality in our database, and reaches a minimum of 1 over 3200 in 1950. Afterwards, the ratio goes up again due to fewer people in the database: as people tend to become famous later in their career, the most recent years (after 1990) have by construction only relatively young individuals who aren't identified yet but will enter the database in the coming decades.

We next run a regression of the log of the ratio of the population in the database to the world population over time. More precisely, denoting by t the calendar time and $lnX(t)$ the log defined above in each year, we estimate

$$lnX_t = a + b \times (2018 - t)$$

The coefficient b is negative and tells us how an additional year of distance to present times leads to a percentage decline in the number of famous people relative to the world population at that time. We find on the restricted dataset that $b = -.0016465$ with a s.d of $.0000146$. The rate at which the fraction of famous people declines after T periods is therefore $1-(1-b)^T$ which is 15.2% each century, or 56.1% after 500 years, or 80.8% after 1000 years.

Figure S1. **Time evolution of GDP, world population and population in the database**

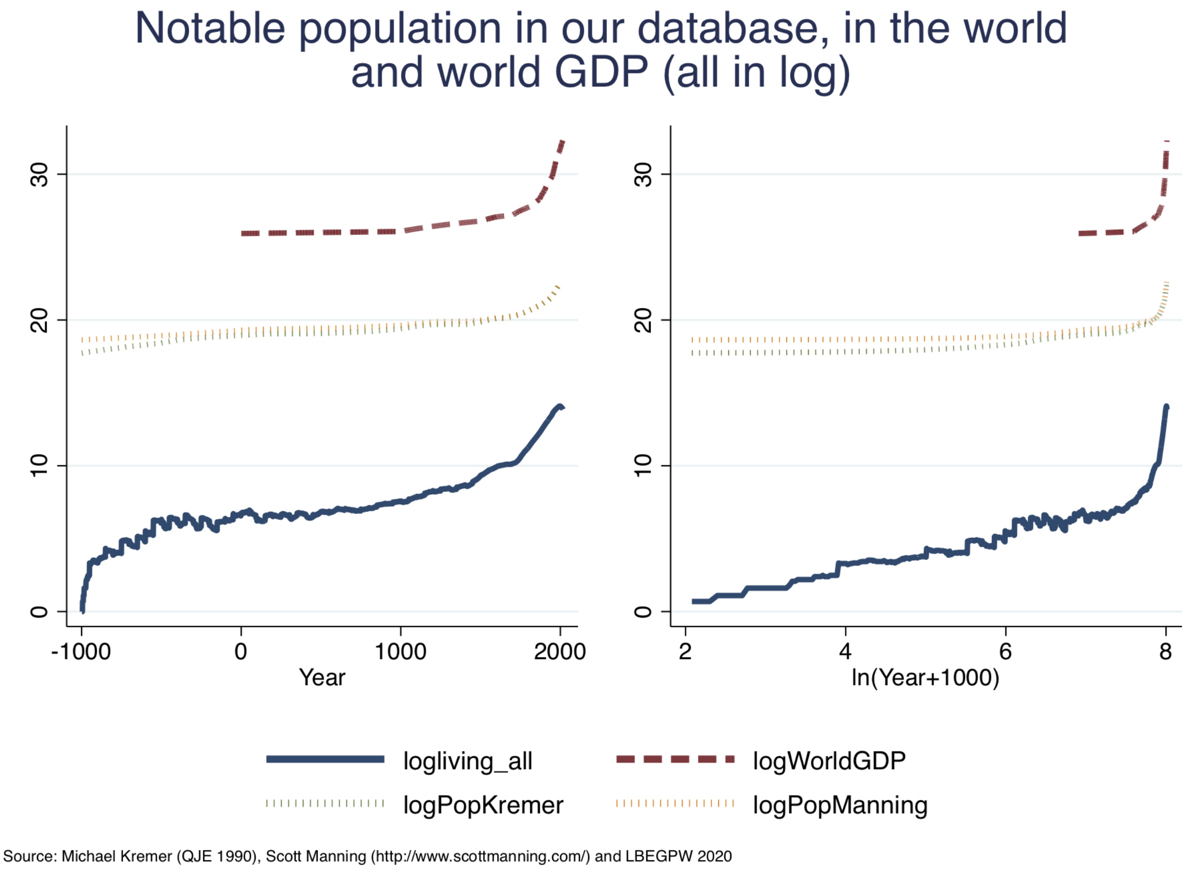

Notes. Restricted sample (at least one `Wikipedia` edition among the 7 European languages analyzed), see Section *Extracting biographic information from a restricted sample*. For a given year, the number of living individuals is calculated by summing up all individuals such that $birth\_date \leq year \leq death\_date$. When not available, the date of birth (resp. death) is estimated from the estimated average longevity over the period. Logliving_all represents the log of the number of alive individuals in the database, logPopManning is the log of estimated world population[7], logPopKremer is the log of estimated world population[6] and logWorldGDP is the log of the estimated world GDP[6]. Left panel: linear scale; right panel: log scale.

Figure S2. **World population relative to the number of notable individuals**

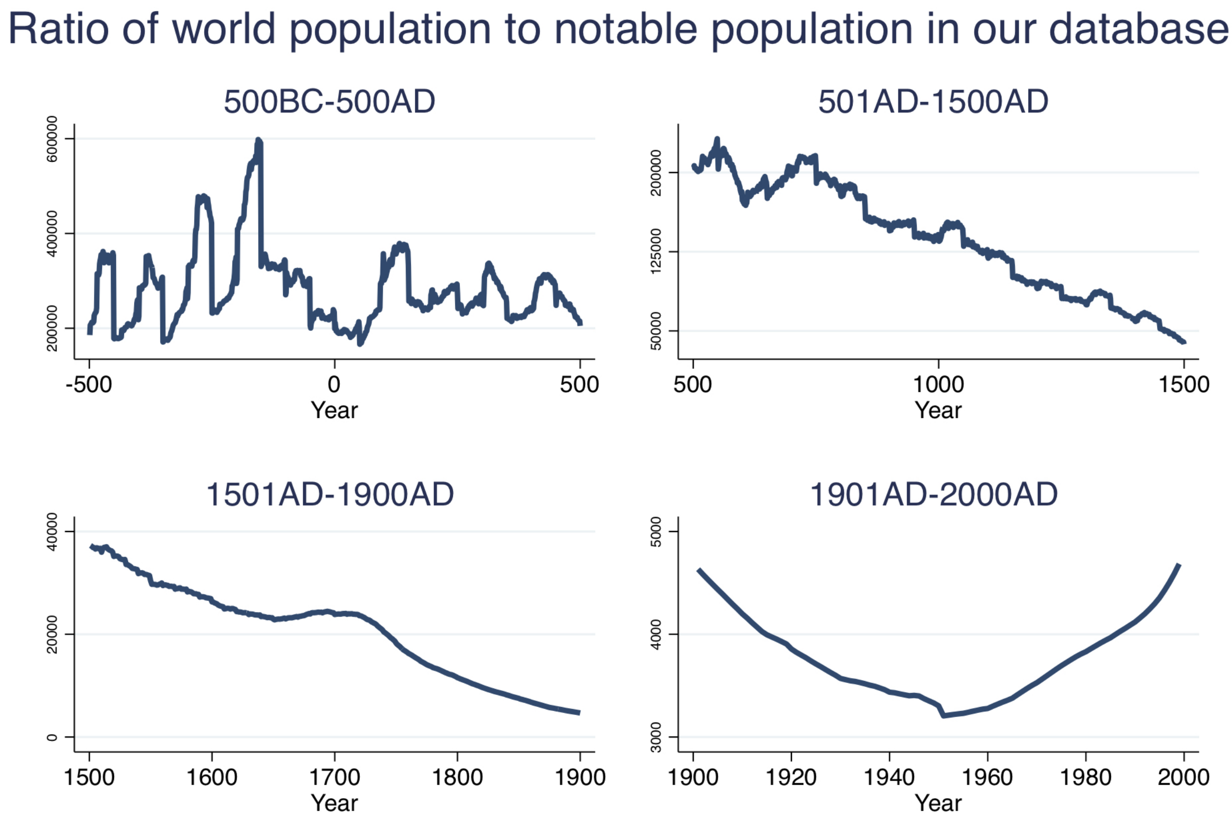

Source: Michael Kremer (QJE 1990), Scott Manning (http://www.scottmanning.com/) and LBEGPW 2020

Notes. Restricted sample (at least one `Wikipedia` edition among the 7 European languages analyzed), see Section *Extracting biographic information from a restricted sample.* For a given year, the number of living individuals is calculated by summing up all individuals such that $birth\_date \leq year \leq death\_date$. When not available, the date of birth (resp. death) is estimated from the estimated average longevity over the period.

A.5 Structure of the database across language editions in `Wikipedia`

In this section, we describe the recursive structure of the database. We list, following an iterative elimination process, the most popular `Wikipedia` editions in decreasing order. For instance, once all individuals with a biography in the English edition have been removed we find 340,913 individuals absent from this edition but present in the German edition, of which 259,013 have a unique biography in this language, etc.

Table S2. Marginal contribution of each language edition

1-25 top language editions				26-50 next language editions			
Edition	Region	#unique	#total	Edition	Region	#unique	#total
English	En	663 930	1 579 940	Slovenian	We	12 193	12 902
German	We	259 013	340 913	Lithuanian	We	11 941	12 305
Japanese	Ea	157 707	179 466	Azerbaijani	EuAr	11 499	12 366
French	We	114 820	155 391	Persian	EuAr	10 441	10 888
Russian	EuAr	98 514	156 728	Romanian	We	10 434	10 644
Chinese	Ea	90 896	97 177	Greek	We	9 699	10 046
Polish	We	86 789	98 407	Indonesian	Ea	9 304	10 883
Spanish	We	72 013	97 540	Esperanto	We	7 953	8 125
Italian	We	63 944	70 263	Armenian	EuAr	7 795	7 852
Swedish	We	56 578	65 719	Kazakh	EuAr	7 178	7 213
Dutch	We	46 215	50 114	Thai	Ea	7 003	7 276
Portuguese	We	43 351	44 446	Galician	We	6 817	6 921
Ukrainian	We	36 886	38 003	Vietnamese	Ea	5 659	5 671
Finnish	We	30 909	31 715	Serbian	We	5 162	6 804
Czech	We	26 670	29 073	Slovak	We	4 693	4 707
Catalan	We	25 866	27 103	Croatian	We	4 484	6 861
Arabic	EuAr	25 585	29 227	Basque	We	3 911	3 935
Korean	Ea	25 000	25 377	Albanian	We	3 791	3 812
Hungarian	We	24 531	33 334	Luxembourgish	We	3 536	3 588
Norwegian (Bokmål)	We	23 433	26 944	Malay	Ea	3 237	4 029
Hebrew	EuAr	17 915	18 540	Belarusian	We	3 174	4 547
Bulgarian	We	17 729	19 847	Latvian	We	3 156	3 198
Estonian	We	15 443	15 793	Haitian	We	2 927	2 933
Danish	We	14 769	14 983	Tagalog	Ea	2 700	2 756
Turkish	EuAr	13 248	14 020	Hindi	Ea	2 573	2 883

51-75 next language editions				76-100 next language editions			
Edition	Region	#unique	#total	Edition	Region	#unique	#total
Afrikaans	South	2 322	2 333	Bashkir	EuAr	519	521
Telugu	East	2 045	2 056	Swahili	South	503	505
West Frisian	West	2 034	2 055	Tongan	South	503	504
Georgian	EuAr	1 992	2 024	Bosnian	West	490	493
Welsh	West	1 865	1 905	Burmese	East	403	404
Marathi	East	1 729	1 734	Chuvash	EuAr	367	370
Tatar	EuAr	1 723	1 844	Kurdish	EuAr	347	364
Bengali	East	1 633	1 652	Piedmontese	West	304	319
Icelandic	West	1 601	1 602	Amharic	South	300	301
Kirghiz	EuAr	1 590	1 604	Alemannic	West	272	278
Norwegian (Nynorsk)	West	1 541	1 544	Occitan	West	262	265
Tamil	East	1 508	1 517	Faroese	West	261	265
Volapük	West	1 366	1 369	Scots	West	255	256
Sakha	EuAr	1 360	1 365	Central Bicolano	East	224	226
Macedonian	West	1 210	1 214	Asturian	West	216	217
Urdu	East	1 085	1 154	Nepali	East	210	243
Tajik	EuAr	914	923	Yiddish	EuAr	210	211
Low Saxon	West	732	733	Gujarati	East	208	214
Latin	West	611	619	Pashto	EuAr	183	185
Mongolian	East	591	592	Aragonese	West	181	182
Breton	West	587	596	Malagasy	East	181	184
Cantonese	East	570	571	Irish	West	175	181
Uzbek	EuAr	566	570	Sicilian	West	164	166
Oriya	East	557	559	Limburgish	West	133	136
Walloon	West	523	535	Scottish Gaelic	West	128	129

Notes. Exhaustive sample (4.7 million individuals). The acronyms *We, Ea, EuAr, Sn* are defined in Table 1, and correspond to groups of language edition of `Wikipedia`. Numbers in this table slightly differ from numbers in Table 1 in that these are based on language editions as per `Wikidata`. These language groups are not linguistic groups, but geographic groups. In Table 1 instead, we used language editions as they appear in `Wikipedia` biographies, which is more relevant for our data extraction based on the 7 language editions of `Wikipedia`. In addition, English in this table includes Old English and Simplified English.

We also report in Table S3 the most famous individuals in the different language editions in recursive order, e.g. an individual in the Eastern language edition does not have a biography in English nor in any of the Western language group.

Table S3. **Visibility index: top 5 individuals in each recursive language group**

`Wikipedia` *(recursive lang. groups)*	
English	Barack Obama, Donald Trump, Leonardo da Vinci, Adolf Hitler, Albert Einstein
Western	Blas de Otero, Anita Blonde, Olivier Nakache, Kristina Rose, Sophie Dee
Eastern	Qays Ibn al-Mulawwah, Husain Waiz Kashifi, Gorō Kishitani, Ryō Iwamatsu, Miyu Takeuchi
Eurasia - Arabia	Aşık Paşa-yı Velî, Erdal Tosun, Roma Acorn, Georgiy Mirskiy, Qayum Nasıyri
Southern and natives	Boerneef, Pieter Pieterse, Jan Blohm, Frank Rautenbach, Tolla van der Merwe
`Wikidata` *only*	Martin Hardie, Lilly Wachowski, John Charles Robinson, Caspar Luyken, Ernest Henri Griset

Notes. Most famous individuals by recursive language group, e.g. absent from above language groups, exhaustive database.

A.6 Data collection: More details (from text)

BIRTH and DEATH dates

When the mode of birth or death in different editions of `Wikipedia` differs from the information in `Wikidata`, we give more credit to the information originating from `Wikipedia` if the information from `Wikidata` is an approximated date, and the reverse otherwise. See for instance Table 4 for detailed statistics of discrepancies between sources. For a significant number of individuals especially from ancient times, the exact year is not available. We then use the century, millennium, circa or decade information when available to estimate it. We build the relevant time intervals and use the middle of the interval as a proxy for birth/death year. Overall, the exact date of birth (death) is known for more than 90% of cases (see Table 3 for exact numbers), and we are able to impute 4% of new birth dates and 14% additional death dates. When the information is available for either birth or death dates, we estimate longevity for the time period, gender, domain of influence and region, and predict the missing date of birth or death based on estimated longevity. When we have no information on both birth and death dates, no imputation is possible and we exclude individuals from all graphs with a time dimension, although many of them are from the 20th and 21st century. Table S1 in Appendix A.4 reports the list of the eldest people in the exhaustive database.

138 **A.7 Evolution of the sample size over time in the restricted and exhaustive databases**

Figure S3. **Time evolution of the number of individuals in the database by gender and language editions**

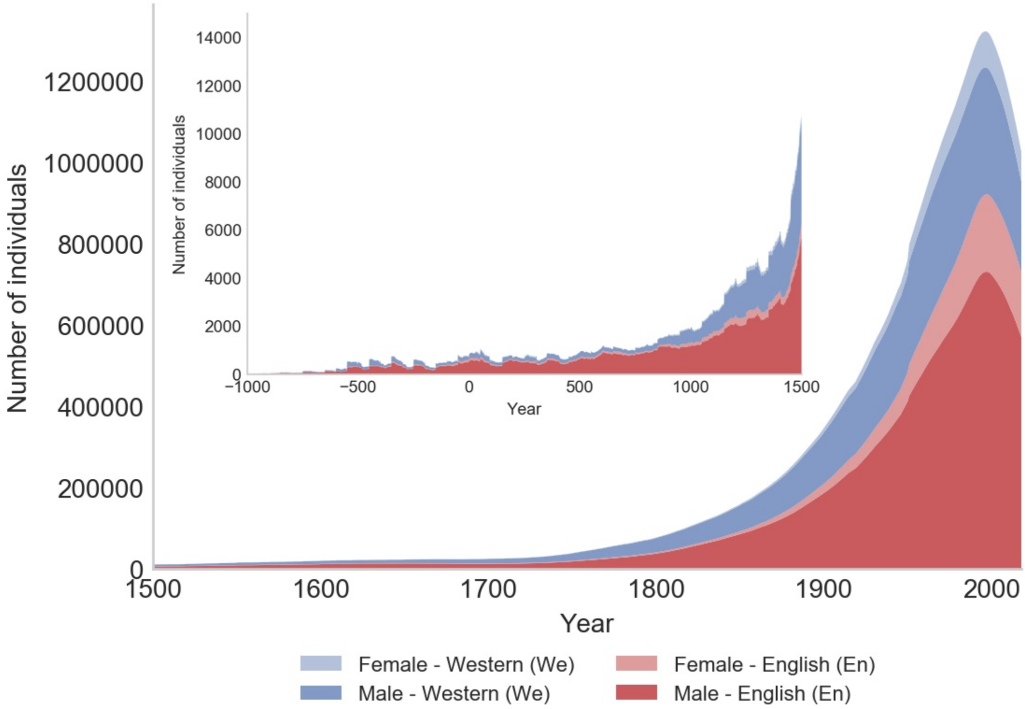

Notes. Cross-verified, restricted sample (at least one `Wikipedia` edition among the 7 European languages analyzed), see Section *Extracting biographic information from a restricted sample*. For a given year, the number of living individuals is calculated by summing up all individuals such that $birth\_date \leq year \leq death\_date$. When not available, the date of birth (resp. death) is estimated from the estimated average longevity over the period. **English (*En*)** language groups include individuals with at least one biography in English in `Wikipedia`; **Western non-English (*We*)** includes individuals with a `Wikipedia` biography in at least one of the Western languages but absent from *En*. See Table 1 for precise definitions of these groups and sub-groups. Individuals with more than one biography account for one observation to avoid double counting.

Figure S4. **Time evolution of the number of individuals in the database by language editions**

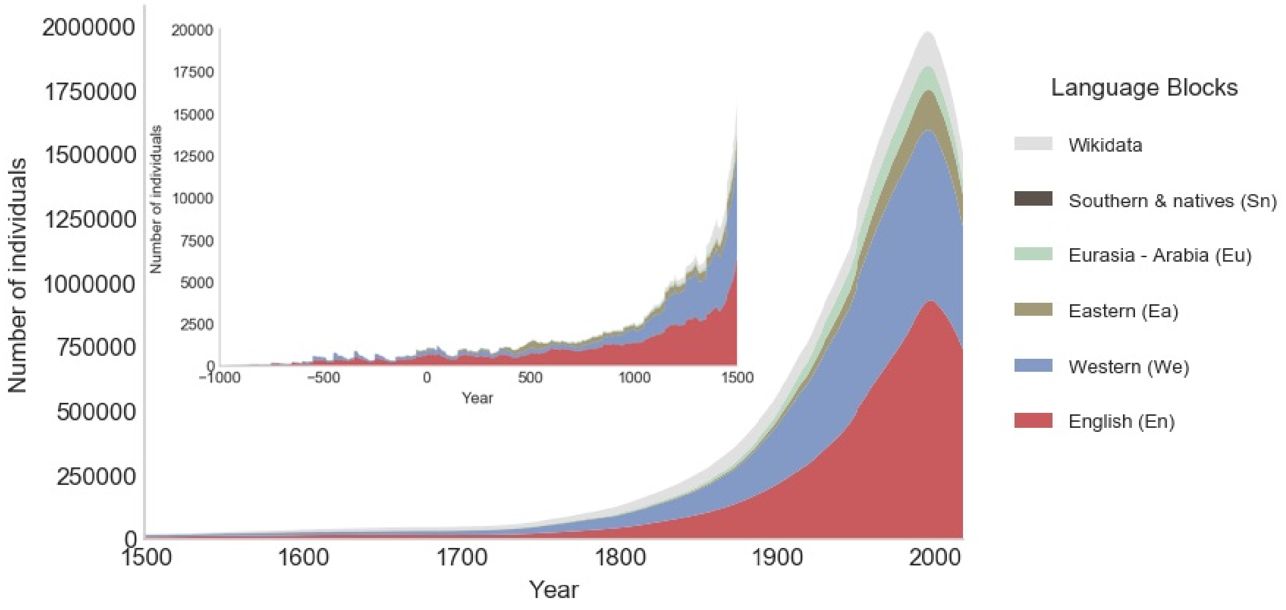

Notes. Exhaustive database. For a given year, the number of living individuals is calculated by summing up all individuals such that $birth\_date \leq year \leq death\_date$. When not available, the date of birth (resp. death) is estimated from the estimated average longevity over the period. English (*En*): individuals present in the English edition; Western (*We*): individuals absent from the *En* edition but present in *We* editions; Eastern (*Ea*): individuals absent from the *En* & *We* groups but with at least one biography in editions of the *Ea* group; Eurasia-Asia (*EuAr*): individuals absent from the previous groups (*En*, *We*, *Ea*) but present in at least one *EuAr* edition. Southern & natives (*Sn*): individuals absent from the other groups (*En*, *We*, *Ea*, *EuAr*) but present in at least one edition of the *Sn* group. **`Wikidata` only** includes individuals with a `Wikidata` biography only. See Table 1 for precise definitions of these groups and sub-groups. Individuals with more than one biography account for one observation to avoid double counting.

Figure S5. **Cloud of the most famous individuals in the database**

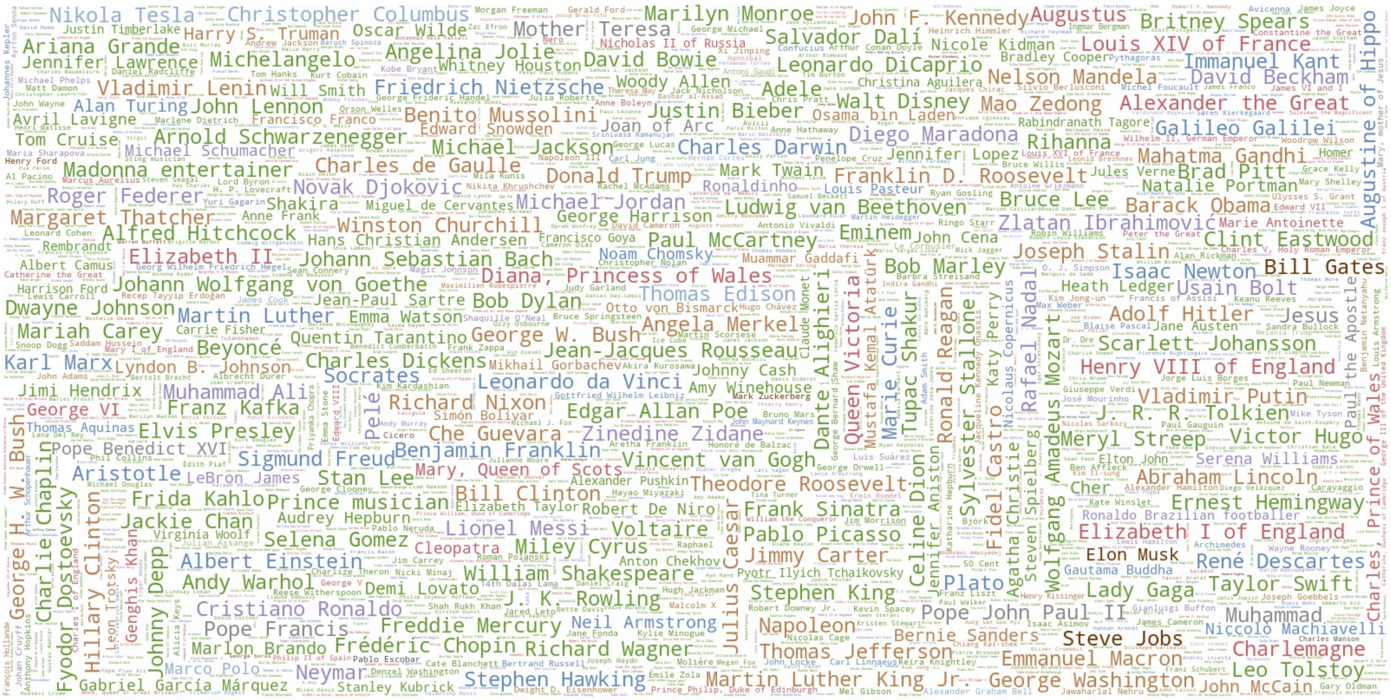

Notes. Size is proportional to relative notability level. The cloud focuses on the 3,000 most visible individuals (0.06% of the exhaustive sample). Colors represent the domain of influence defined in section (Domains of influence and occupations) (see Figures 1 and 2 for labels, e.g. green is culture, red is politics, blue is academia, etc.).

Figure S6 shows the dispersion across individuals in this notability index. The chart shows the distribution by recursive language group. With no surprise, the English edition contains relatively more visible individuals than the other language groups. For the Western language group, the distribution is bi-modal, both peaks being quite low in terms of notability with respect to the overall distribution of notability and dominated by individuals from the English edition. For the other language groups, the notability index is even lower.

B Data processing for the restricted sample

B.1 Details on the allocation into domains of influence

DOMAINS OF INFLUENCE

In `Wikipedia`, keywords related to the domain of influence are found in the first part of most biographies after verbal groups such as "was a"/"is a"/"was the"/"is the". We first parse the English edition to detect keywords in a list of 1911 occupations and select the first three keywords. In most cases, these correspond to a well-referenced occupation such as pianist, engineer, politician, general, etc. To give an example, we collected three different keywords for Ray Charles from the following part of sentence: "*Ray Charles was an American singer, songwriter, musician*". We also consider the other language editions using the set of keywords extracted from the English edition translated into French, Spanish, Italian, Swedish, German and Portuguese. The maximum number of keywords collected for a single individual is 21 (3 keywords per edition). We did not find any keywords for 44,808 individuals (2% of individuals with a `Wikipedia` biography). `Wikidata` also contains information about those domains of influence. On average, we find 1.3 occupation per `Wikidata` entry; 99.5% individuals have less than 6 occupations. Most of the time, the number of reported occupations is one. In this universe, Ray Charles: `Wikidata` is classified as *musician, singer, composer, pianist, singer-songwriter, saxophonist, vocalist, arrangement, jazz musician*. We group the 1911 identified keywords in five large categories of occupations, and a sixth residual category. We also split categories into sub-categories as follows (in parenthesis we report how they match with the theoretical concepts of section): First, we determine the most recurring sub-category (mode) from either `Wikipedia` and `Wikidata` and compare them. We get a match in most cases. See for instance Table 4 for the systematic comparison of sources. In case the information is missing in one universe, we use the sub-category found in the other source when available. When sub-categories are available in both sources, we give a preference to `Wikidata`, under the assumption that this repository is more structured and less subject to errors. Moreover, we report a second domain of influence (the second most frequent one), based on the full list of domains identified from `Wikidata`

Figure S6. **Density distribution of notability by language groups**

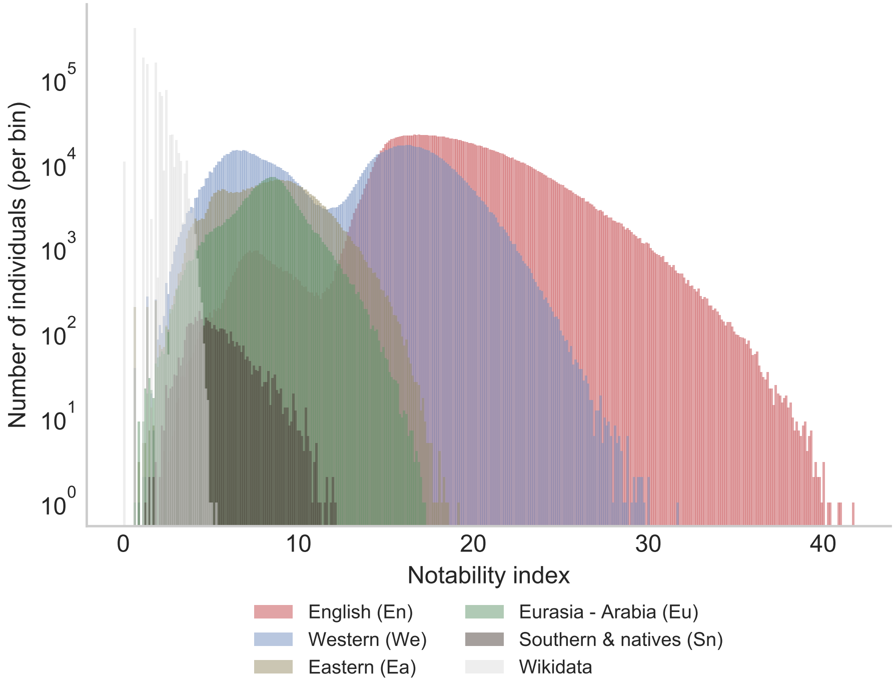

Notes. Exhaustive sample (4.678 million individuals). English (*En*): individuals present in the English edition; Western (*We*): individuals absent from the *En* edition but present in *We* editions; Eastern (*Ea*): individuals absent from the *En* & *We* groups but with at least one biography in editions of the *Ea* group; Eurasia-Asia (*EuAr*): individuals absent from the previous groups (*En*, *We*, *Ea*) but present in at least one *EuAr* edition. Southern & natives (*Sn*): individuals absent from the other groups (*En*, *We*, *Ea*, *EuAr*) but present in at least one edition of the *Sn* group. **`Wikidata` Only** includes individuals with a `Wikidata` biography only.

and all `Wikipedia` pages. We use a frequency threshold equal to 25% above which we keep the second occupation. This threshold value has been determined in a pilot study in which we gauged the number of errors generated when using more or less constraining threshold values. A good illustration is Napoleon Bonaparte who is referenced in two main domains: “Politics” and also “Military”. Another example is Ronald Reagan, famous first for his prominent role in American Politics in the 80’s and also known as an actor.

To sum up, the easy cases are when `Wikipedia`’s and `Wikidata`’s keywords characterizing an occupation or a domain of influence converge towards two identical modal occupations across sources. When this information diverges, we generally give more credit to `Wikidata`. We however make an exception to this rule when there is a tie between the modes in `Wikidata` and instead a clear, unique, mode in `Wikipedia`. In this case, we favor `Wikipedia`. In the more problematic case in which both `Wikidata` and `Wikipedia` give several modes, we pool all keywords together and determine the mode from this combined list.

B.2 Details on the definition and creation of citizenships

CITIZENSHIP

The level of agreement across `Wikidata` and `Wikipedia` on citizenship is at around 95%. In case both sources contradict each other on this dimension, we give more credit to the information contained in the Infobox (`Wikipedia`) and `Wikidata`. For most citizenships, we make a distinction between "old regime" and "current regime" and use the acquisition of sovereignty information to determine whether an individual’s citizenship belongs to one or the other. We proceed the same way with empires to correctly assign individuals to either these supranational entities or to the new nation states that emerged after their collapse. In the matter, we consider the three supranational entities: Holy Roman Empire, Roman Empire and Soviet Union. This grouping procedure was made necessary given both the geographical expanse of such political entities and the fact that it is almost impossible to associate them with a single modern country. Finally, a fraction of our individuals have several citizenships and we report two of them whenever appropriate.

Next, we match citizenships and political entities at the time of the individuals life, using information on the creation of modern states to determine whether we should assign the individuals to the new or the old regime of the country. The old regimes also encompass all political entities broadly situated in the current geographical location of the modern state. For e.g.: Erstwhile

colonies under the British empire such as India get divided under Old regimes of the country (India) vs India based on their independence date. The Mughal Empire, the Chola and Chela Kingdom get classified under Old regimes of India too. In cases where both birth and death dates of the individual are before (after) the date of foundation of the modern state, we assign the individual to old regime (modern regime). When the birth date is before and the death date is after the foundation of the modern state, we assign the individual to the new regime if and only if the modern state was explicitly mentioned as one of the citizenships in the disaggregated information collected from `Wikidata` in the first step, otherwise we assign her to the old regime. The citizenship for individuals assigned to the old regime reads as Old_(before_year_xx)_YY where xx refers to the threshold year used to demarcate the old regimes from the modern state and YY refers to the name of the modern state. For instance, Akbar's (the Mughal emperor) citizenship would read as Old_(before_year_1947_AD)_India.

As for occupations, the easy cases are the ones where the information on citizenship from `Wikidata` and `Wikipedia` match. When they instead contradicted each other, we retain information from `Wikipedia` if and only if it matches with a time invariant citizenship from `Wikidata`; or if the information obtained from `Wikipedia` is present in the Infobox of at least one language edition scraped; otherwise we assign the citizenship from `Wikidata`. The reason why we give more credit to `Wikidata` in the other cases is that the code written to extract this information in `Wikipedia` may introduce more mistakes, since it needs to crawl the entire content of the biography to detect one or several citizenships that do not necessarily belong to the individual. Lastly, in case the citizenship information is absent from one universe, we use the most frequent citizenship(s) found in the other universe.

A large number of individuals have two citizenships, either because they are true bi-nationals (e.g. Indian and US citizens) or because the country they were born in, disappeared or separated from a larger entity (for example, Bosnia and Herzegovina from Yugoslavia in the 90's). We therefore decide to report up to two citizenships in the database for a better coverage.

The thresholds used to demarcate old political and geographical regimes from the modern state for each nation state are available at: https://en.wikipedia.org/wiki/List_of_sovereign_states_by_date_of_formation.

B.3 Sources of information

The restricted sample is clearly dominated by the German edition which is derived from two main sources: i) VIAF and ii) the Deutsche National Bibliothek. The latter source is not intensively used in the other `Wikipedia` editions, while the former is often mentioned in the main `Wikipedia`. One learns from these graphs that the efforts to inflate the number of biographies in some editions would require the construction and development of national repositories.

Figure S7 represents the correspondence between editions and source of information related to individuals. The left panel represents the exhaustive sample while the right panel is restricted to individuals with one unique biography in the `Wikipedia` universe. Only a small fraction of biographies (11.6%) does not report any specific source. In the matter, the most frequent sourced mentioned are, in decreasing order: the Virtual International Authority File (VIAF) (the VIAF is a joint project of several national libraries operated by the Online Computer Library Center (OCLC)), `Freebase`, the Deutsche National Bibliothek, followed by two French sources (Bibliotheque Nationale de France and a French collaborative library catalogue). One can see that the large number of German individuals stems from two main sources, the VIAF and the Deutsche National Bibliothek. The latter source does not provide many links to other `Wikipedia` editions, while the former also brings individuals with a single biography in English.

B.4 Additional trends

B.4.1 Arts: the Quattrocento and the Dutch Golden Age

We also expand the coverage of notable individuals in the arts sector. Two important artistic movements clearly stand out from the left panel of Figure S8. The Italian Quattrocento first, which corresponds to the left part of the large green area, that dominates most of the period ranging from the early 15th century till approximately the beginning of the 18th century. The Quattrocento was one of the most important periods of European art and culture. It is referred to the first phase of the movement known as Renaissance. This period is followed by three other important periods in Italian Art history: Cinquecento (1500s), Mannerism (1527 to 1580), Baroque (1600 - 1750) and Rococo Art (1699 - 1780). In the matter, the contribution of the Italian edition is sizeable. The vertical bars in the right panel show that more than 95% of Italian painters were added and absent from existing databases.

The second period corresponds to the Dutch Golden Age which competes with the Italians all over the seventeenth century. The Golden Age in Dutch History is a period spanning from 1581 to 1672, in which Dutch trade, science, and art and the Dutch military were ranked among the most powerful and influential in the world. The first part of the period analyzed is characterized by the Eighty Years' War, which ended in 1648. The number of individuals added with a Dutch citizenship who are not in the English edition of `Wikipedia` is larger than 90%.

At the end of the period, we acknowledge the rise of US modern painting. Here again, extending the scope of the database to less-known individuals proves quite useful. Examples of famous individuals who make their first appearance in a knowledge base are many. Pietro Paolo Vasta (https://it.wikipedia.org/wiki/Pietro_Paolo_Vasta) (1697-1760, Painter) is an Italian painter and one of the most emblematic renowned member of Sicilian Baroque movement which evolved on the island of Sicily.

Figure S7. Relation between the most frequent `Wikipedia` editions and sources

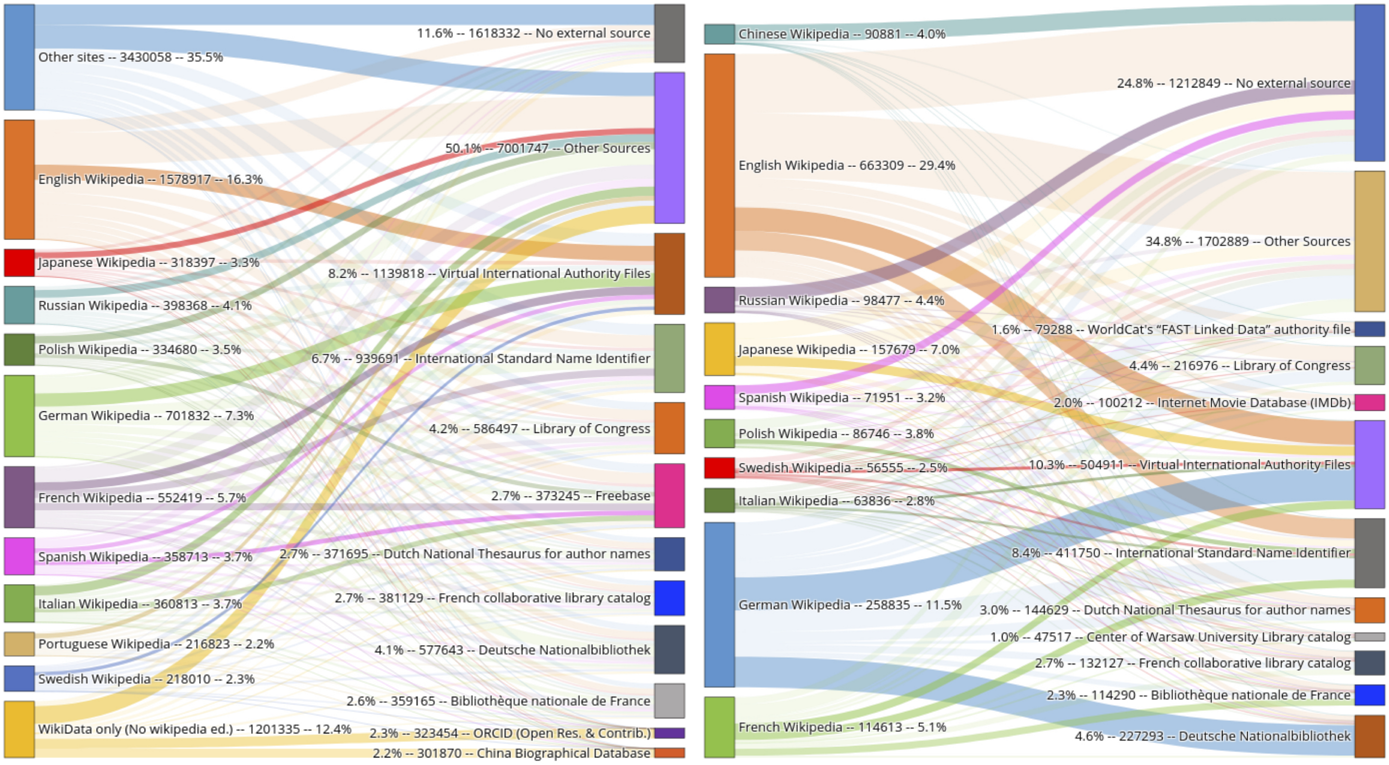

Notes. Exhaustive sample (left panel) and sample restricted to individuals with only one biography in `Wikipedia` (right panel). We consider the 10 most frequent external sources and 10 most popular `Wikipedia` language editions, and merge the rest into other sources.

Figure S8. Evolution of the share of individuals per citizenship identified as "painter", 1400-2000AD

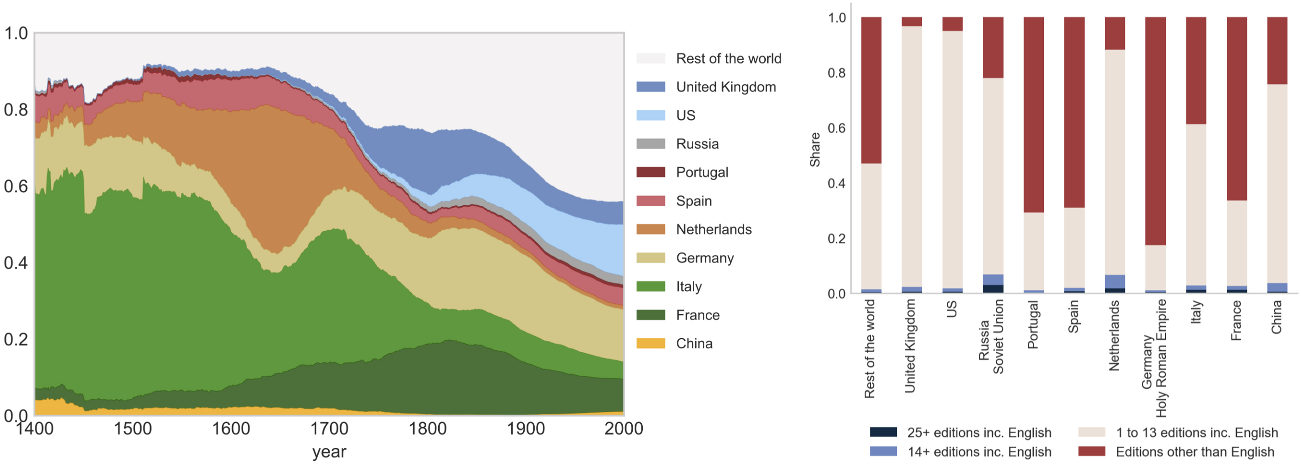

Notes. Cross-verified, restricted sample (at least one `Wikipedia` edition among the 7 European languages analyzed), see Section *Extracting biographic information from a restricted sample*. The occupations are defined in Section *Domains of influence and occupations*. For a given year, the number of living individuals is calculated by summing up all individuals such that $birth\_date \leq year \leq death\_date$. When not available, the date of birth (resp. death) is estimated from the estimated average longevity over the period. Left panel: Most popular citizenships (share of living individuals on the vertical axis, year on the horizontal axis). Right panel: `Wikipedia` profile: share of individuals with biographies in a) 25+ editions including English, b) 14+ editions including English, c) 1 to 13 editions including English, d) One or more editions than English), breakdown by citizenship.

B.4.2 *Religion and theologians: Hegira and Reformation*

Figure S9 shows the cumulated number of *theologians* in the restricted sample. Three patterns emerge: the rise of Islamic theologians in the fertile crescent following the Hegira in 622, the steady growth of Christian theologians in Europe until the break related to the Protestant reform (1517). After that period, the number of Protestant theologians rose at an exponential rate in the restricted sample. The role played by the additional editions we considered is important here as well as shown by the large red bars for most citizenships. This brings information about this specific category of notable individuals who played a central role in the History of civilizations.

Figure S9. Evolution of the number of individuals identified as "theologian", 100-2000AD

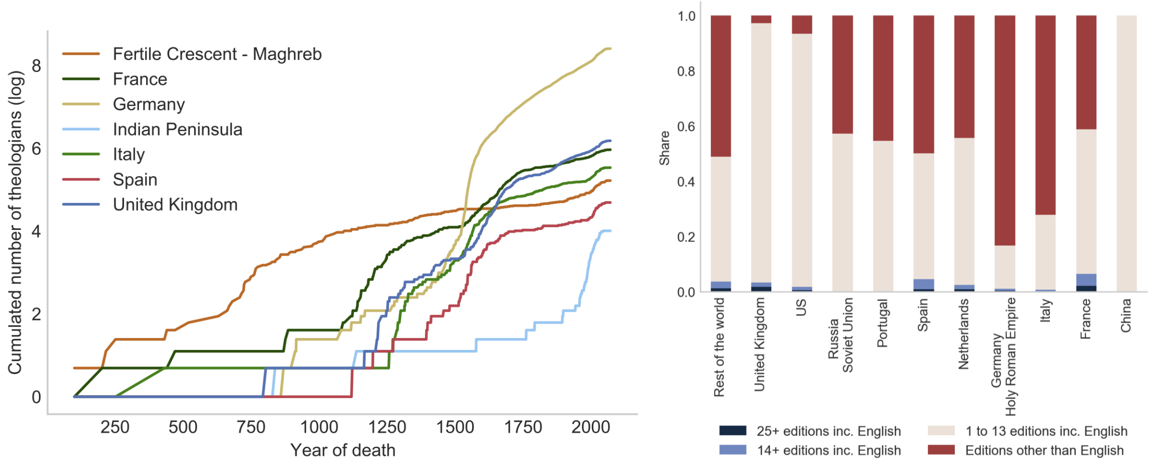

Notes. Cross-verified, restricted sample (at least one `Wikipedia` edition among the 7 European languages analyzed), see Section *Extracting biographic information from a restricted sample*. The occupations are defined in Section *Domains of influence and occupations. For a given year, the number of living individuals is calculated by summing up all individuals such that* $birth\_date \leq year \leq death\_date$*. When not available, the date of birth (resp. death) is estimated from the estimated average longevity over the period.* Left panel: number of theologians (number of living individuals on the vertical axis, year on the horizontal axis). Right panel: `Wikipedia` profile: share of individuals with biographies in a) 25+ editions including English, b) 14+ editions including English, c) 1 to 13 editions including English, d) One or more editions than English), breakdown by citizenship, y-axis in natural logs.

B.4.3 *Longevity: war and peace*

The evolution over time of median longevity is shown in Figure S10. It was computed as the difference between death year and birth year when available. As in previous studies[5], we observe steady improvements in longevity of the cohorts born after 1600. However here we do not observe noticeable differences across language editions (left panel). The evolution over time of median longevity is lower for individuals in military and nobility domains (right panel), compared to academia and religious domains. Concerning nobility, the death of noble infants drives down the median life expectancy.

Figure S11 represents the age at death for individuals in the sample on the period 1700-1960. On the left panel, war episodes are noticeable as darker downward sloping lines corresponding to abnormal death rates during war periods with even darker points for young generations further exposed in those conflicts (from the right to the left, WWII, WWI, the American civil war, etc.). A detailed timeline of historical events based on a similar change in age of death can be found in the literature[8] (see their Figure 4, page 561), with a comparison of `Ngrams` intensity per period and the frequency of death. One observes the trace of the American civil war on the left chart but not on the right chart. Instead, one observes a small trace on the right around 1936 which corresponds to the Spanish civil war.

B.5 Summary: notes on the differences between the English and the Western (non-English) editions

The content of existing databases on notable individuals have so far been compiled from the English edition of `Wikipedia` exclusively. Working with the English edition was back then, quite a natural choice as English is still, to date, the largest edition in the `Wikipedia` universe with 1,579,940 different biographies. In this section, we tried to provide details on the addition of non-English editions.

Taking stock, Table S4 provides basic descriptive statistics based on two different samples for birth date, domain of influence, gender and citizenship: a) individuals present in the English edition (and possibly in other editions) and b) individuals absent from the English edition that we call here the Western non-English sample.

It is interesting to note that individuals in the English sample were born on average more recently than those included in the Other editions sample (first three columns). Their main domains of influence are also quite different. Sports, for example,

Figure S10. Longevity, 1000BC-2000AD

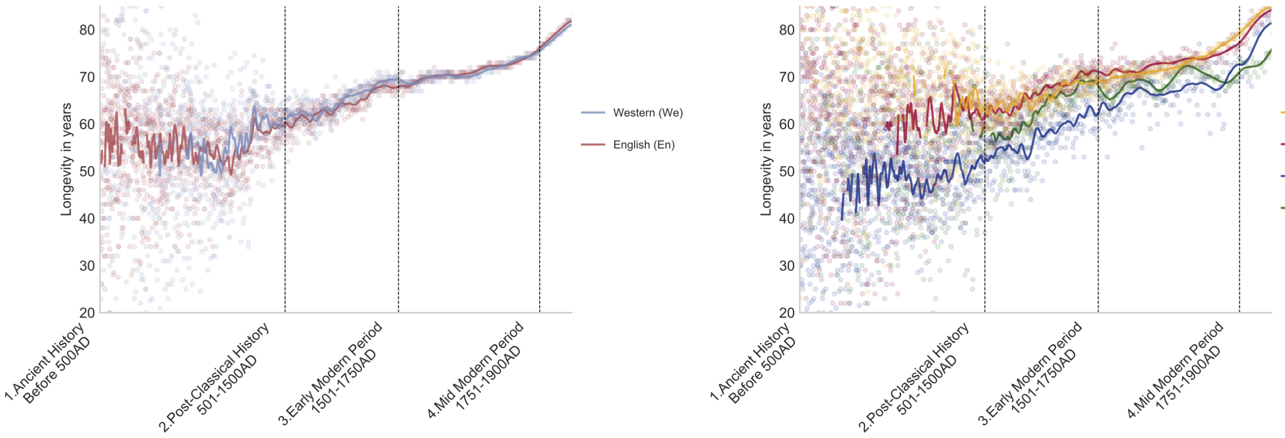

Notes. Cross-verified, restricted sample (at least one `Wikipedia` edition among the 7 European languages analyzed), see Section *Extracting biographic information from a restricted sample*. The occupations are defined in Section *Domains of influence and occupations. For a given year, the number of living individuals is calculated by summing up all individuals such that* $birth\_date \leq year \leq death\_date$*. When not available, the date of birth (resp. death) is estimated from the estimated average longevity over the period.* Dots correspond to median life expectancy at birth, solid lines represent moving average over 20 years when observations are available. The following log transformation has been applied to the time axis: $year \rightarrow 8.5 - log(2019 - year)$

Figure S11. Age at death on English (left) and Western non-English editions (right), 1800-2000AD

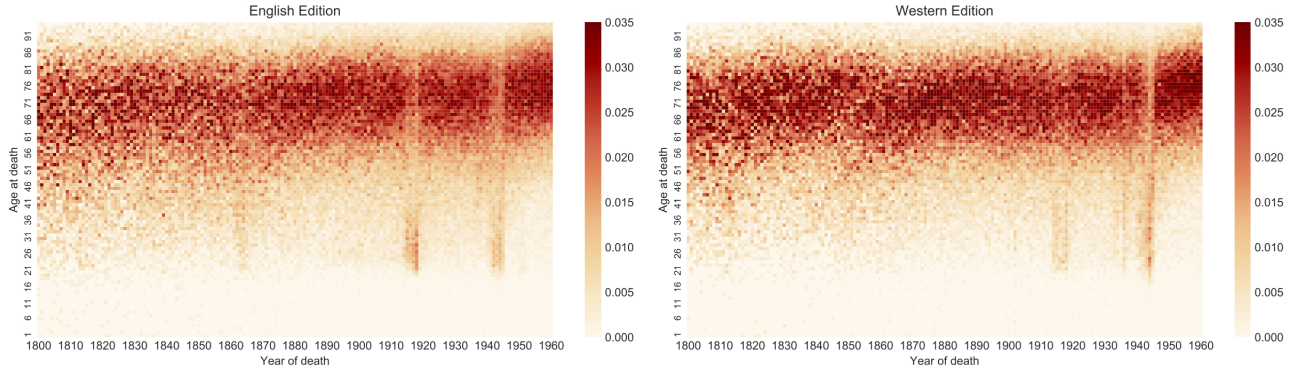

Notes. Cross-verified, restricted sample (at least one `Wikipedia` edition among the 7 European languages analyzed), see Section *Extracting biographic information from a restricted sample*. The occupations are defined in Section *Domains of influence and occupations. For a given year, the number of living individuals is calculated by summing up all individuals such that* $birth\_date \leq year \leq death\_date$*. When not available, the date of birth (resp. death) is estimated from the estimated average longevity over the period.* In both panels, a vertical line corresponds to the distribution of the age at death for a given date. The observed colors discontinuity illustrates wars episodes: American Civil War, First World War, Spanish Civil War, and Second World War.

is more prevalent in the English sample while Culture dominates in the Western non-English sample. The fraction of female individuals varies marginally. It is slightly higher in the English sample (18%) than in the other editions sample (15%). The most popular citizenships detected in the English sample are American, UK, Canadian, French versus German, French and Swedish in the Western non-English sample.

Results:

1. Historical periods specific to a country are quantitatively better covered by the addition of the Western non-English edition. The French edition of `Wikipedia` allows to better document the emergence of politicians during the French Revolution; the German edition allows to better document the German reformation and the emergence of the Prussian empire, the Spanish and Portuguese editions improve the coverage of the Age of Explorations.
2. The granularity of the database allows to focus on rare occupations such as theologians or on events such as the emergence of journalism.

3. The share of women is substantially higher in the Western non-English editions of `Wikipedia` after 1950, but overall 1.4 percentage point below.
4. The American Civil War is visible in the English edition but not in the Western non-English edition, and the contrary holds for the Spanish civil war.
5. The Western non-English editions focus more on culture and politics and less on sport, and are less centered on individuals from the U.K. and the U.S. and more on Continental Europe, in particular Germany, France and Sweden.

C Test protocol

C.1 Pilot

10 RAs from Sciences Po Paris, NYUAD and Delhi School of Economics received a sample of 1000 individuals to test.

C.1.1 Instructions

- We will give you 1000 individuals from various notability levels, and ask you to check and validate or report mistakes on 6 different pieces of information: exact or approximate date of birth and death; gender; main occupation and possibly secondary occupation; citizenship or equivalent concept for earlier periods of history.
- You will be asked to report the verification in the google sheet next to each information. "Correct" means no error, "Error" means certain error, "Missing" means the information is included in `Wikipedia/Wikidata` but not present in the dataset, "Other case" means possible error. Judgment is required from you.
  - For instance, if there is a historical controversy and several sources differing, report "Other case" unless there is an obvious mistake in our database.
  - It will be particularly the case for the retained citizenship that is sometimes selected among a list of ten or more different geographical areas, kingdom, franchised cities, duchy, caliphate etc., the borders of which evolved during the life of the individual.
  - The information on birth (and death if relevant) is sometimes approximated by $birth\_min$ or $birth\_max$ (by $death\_min$ or $death\_max$). For instance, someone only known for being born in the 12th century will be reported as $birth\_min = 1101$ and $birth\_max = 1201$ and $birth\_b = N/A$
- Description of variables
  - $birth\_b =$ date of birth (exact)
  - $death\_b =$ date of death (exact)
  - $birth\_min\_b$ = minimum date of birth (intervals because approximation)
  - $birth\_max\_b =$ maximum date of birth (intervals because approximation)
  - $death\_min\_b =$ minimum date of death (intervals because approximation)
  - $death\_max\_b =$ maximum date of death (intervals because approximation)
  - $gender\_b =$ gender
  - $final\_occupation =$ 1st final occupation (level 2)
  - $freq\_1stoccu =$ Frequency associated to 1st occupation (level 2)
  - $final\_second\_occup =$ 2nd final occupation (level 2)
  - $freq\_2ndoccu =$ Frequency associated to 2nd occupation (level 2)
  - $keyword\_used =$ keyword used to define 1st final occupation
  - $area1\_of\_ratt =$ 1st Citizenship (distinction current/former country)
  - $area2\_of\_ratt =$ 2nd Citizenship (distinction current/former country)
  - $euro7\_editions =$ availability of 7 European language editions
- Cross-verification: A part of the sample is common to other research assistants to assess the accuracy of your work. There will be an end of contract reward of up to 12.5% of the contract for the quality of the work.
- Remember that the goal is neither to minimize nor to maximize the number of spotted errors but to detect and provide a fair assessment of the quality of the database. Keep all your comments and suggestions on the spreadsheet as it may be requested by editors of scientific journals. In case of doubt, report "Other case" as explained above, and the reason for the doubt about the information contained in the database.

At the end of the pilot, we looked at the various errors detected. In particular, as regards to occupations, we noticed that when the frequency of the second occupation was below 0.25, there was a large proportion of errors; we decided to set this as a threshold, since it preserves many true positive regarding the second occupation.

C.2 Final test

See the text.

C.2.1 Instructions, final set

- We will give you 1000 individuals from various notability levels, and ask you to check and validate or report mistakes on 6 different pieces of information: exact or approximate date of birth and death; gender; main occupation and possibly secondary occupation; citizenship or equivalent concept for earlier periods of history.
- You will be asked to report the verification in the google sheet next to each information. “Correct” means no error, “Error” means certain error, “Missing” means the information is included in `Wikipedia/Wikidata` but not present in the dataset, “Other case” means possible error. Judgment is required from you.
  - For instance, if there is a historical controversy and several sources differing, report “Other case” unless there is an obvious mistake in our database.
  - It will be particularly the case for the retained citizenship that is sometimes selected among a list of ten or more different geographical areas, kingdom, franchised cities, duchy, caliphate etc., the borders of which evolved during the life of the individual.
  - The information on birth (and death if relevant) is sometimes approximated by $birth\_min$ or $birth\_max$ (by $death\_min$ or $death\_max$). For instance, someone only known for being born in the 12th century will be reported as $birth\_min = 1101$ and $birth\_max = 1201$ and $birth\_b = N/A$
- Description of variables
  - $birth\_b =$ date of birth (exact)
  - $death\_b =$ date of death (exact)
  - $birth\_min\_b$ = minimum date of birth (intervals because approximation)
  - $birth\_max\_b =$ maximum date of birth (intervals because approximation)
  - $death\_min\_b =$ minimum date of death (intervals because approximation)
  - $death\_max\_b =$ maximum date of death (intervals because approximation)
  - $gender\_b =$ gender
  - $final\_occupation =$ 1st final occupation (level 2)
  - $freq\_1stoccu =$ Frequency associated to 1st occupation (level 2)
  - $final\_second\_occup =$ 2nd final occupation (level 2)
  - $freq\_2ndoccu =$ Frequency associated to 2nd occupation (level 2)
  - $keyword\_used =$ keyword used to define 1st final occupation
  - $citizenship\_1\_b$ 1st Citizenship (no distinction current/former country)
  - $citizenship\_2\_b$ 2nd Citizenship (no distinction current/former country)
  - $year\_creation\_state1$ and 2: year of the creation of the modern state in $area1\_of\_ratt1$ or 2
  - $euro7\_editions =$ availability of 7 European language Editions.
- Cross-verification: A part of the sample is common to other research assistants to assess the accuracy of your work. There will be an end of contract reward of up to 12.5% of the contract for the quality of the work.
- Remember that the goal is neither to minimize nor to maximize the number of spotted errors but to detect and provide a fair assessment of the quality of the database. Keep all your comments and suggestions on the spreadsheet as it may be requested by editors of scientific journals. In case of doubt, report “Other case” as explained above, and the reason for the doubt about the information contained in the database.

References

1. Postel, H. J. Die kölner phonetik. ein verfahren zur identifizierung von personennamen auf der grundlage der gestaltanalyse. *IBM-Nachrichten* **19**, 925–931 (1969).
2. Pollock, J. J. & Zamora, A. Automatic spelling correction in scientific and scholarly text. *Commun. ACM* **27**, 358–368, https://doi.org/10.1145/358027.358048 (1984).
3. Hernández, M. A. & Stolfo, S. J. The merge/purge problem for large databases. In *Proceedings of the 1995 ACM SIGMOD international conference on Management of data - SIGMOD '95*, 127–138, https://doi.org/10.1145/223784.223807 (ACM Press, San Jose, California, United States, 1995).
4. Levenshtein, V. I. Binary Codes Capable of Correcting Deletions, Insertions and Reversals. *Sov. Phys. Doklady* **10**, 707 (1966).
5. de la Croix, D. & Licandro, O. The longevity of famous people from Hammurabi to Einstein. *J. Econ. Growth* **20**, 263–303 (2015).
6. Kremer, M. Population growth and technological change: One million bc to 1990. *The Q. J. Econ.* **108**, 681–716 (1993).
7. Manning, S. Year-by-year world population estimates: 10,000 bc to 2007 ad. *Hist. on warpath* **12** (2008).
8. Schich, M. *et al.* A network framework of cultural history. *Science* **345**, 558–562 (2014).

Table S4. Sample statistics: breakdown by language edition (English vs Western non-English)

Wikipedia (recursive lang. editions)	Birth year (percentile) 10	50	90	Occupation %	Female %	Citizenship %
English	1821	1946	1988	SP:34,CLT1:24.1,POL:12.8,ACAD:9.1	17.7	US:25.2,UK:13.6,CA:3.9,FR:3.5
Western non-English	1788	1928	1979	CLT1:31.7,POL:15.6,SP:15,ACAD:14.6	15.3	DE_O:14.8, DE:13.3,FR:13,SE:7.6

Notes. Restricted sample (at least one Wikipedia edition among the 7 European languages analyzed), see Section *Extracting biographic information from a restricted sample*. The occupations are defined in Section *Domains of influence and occupations*. This table provides some summary statistics (birth date, domain of influence, share of females, citizenship) on two samples: English versus Non-English. SP = Sports/Games, CLT1 = Culture-Core, POL = Politics, ACAD = Academia, US = United-States, UK = United Kingdom, FR = France, CA = Canada, DE = Germany, SE = Sweden, DE_0 = Germany (Former political/geographical entity).

Table S5. Manual verifications: discrepancy between RAs on each variable

	Birth	Death	Gender	1st Occupation	2nd Occupation	1st Citizenship	2nd Citizenship
Mismatch (# Obs)	142	69	75	207	467	217	128
(%)	2.84	1.38	1.50	4.14	9.34	4.34	2.56

Notes. This table provides the numbers and rates of discrepancy, when independent RAs did not report the same outcomes among Correct, Error, Missing, Other case for the same individual. The first row gives the number and the second row gives the frequency.

Table S6. Manual verifications: summary statistics Sample: mix of sub-samples

	Birth	Death	Gender	1st Occupation	2nd Occupation	1st Citizenship	2nd Citizenship
Information not reported in our database							
No info in sources	12.12	53.45	2.04	3.66	64.13	6.76	92.24
Info. updated since collec.	0.90	0.44	3.42	2.76	2.26	1.02	1.14
Information reported in our database							
Correct	84.68	45.01	94.44	92.50	28.21	91.30	5.96
Error	0.64	0.22	0	1.00	5.04	0.80	0.56
Other case (ambiguity or info. updated since collec.)	1.66	0.88	0.10	0.08	0.36	0.12	0.10
Total # cases	4,999	4,999	4,999	4,999	4,999	4,999	4,999

Notes. Test sample on a mix of the exhaustive and restricted database (at least one Wikipedia edition among the 7 European languages analyzed) with over sampling, see text. This table provides some summary statistics on manual checks. The different possible outcomes are" "No info in sources" means the information is not included in Wikipedia/Wikidata nor in our dataset, "Info updated since data collection" means the information is included in Wikipedia/Wikidata but not present in our dataset; "Information Correct" means no error, "Error" means certain error, "Other case" means possible error (for instance historical controversy, several sources differing or information updated since data collection). These checks have been conducted by the 10 RAs and cross-verified by a PhD researcher.

Table S7. Manual verifications: summary statistics
Sample: 1+ Europ. Wikipedia eds.

	Birth	Death	Gender	1st Occupation	2nd Occupation	1st Citizenship	2nd Citizenship
Information reported in our database							
No info. in sources	7.80	55.10	0	0.27	61.27	1.17	95.10
Info. updated since collec.	0.70	0.37	0.10	0.50	1.43	0.83	1.03
Information not reported in our database							
Correct	89.00	43.50	99.77	97.60	29.97	96.87	3.43
Error	0.87	0.27	0	1.53	6.83	0.97	0.37
Other case (ambiguity or info. updated since collec.)	1.63	0.77	0.13	0.10	0.50	0.17	0.07
TOTAL	3,000	3,000	3,000	3,000	3,000	3,000	3,000

Notes. Test sample on the restricted database (at least one Wikipedia edition among the 7 European languages analyzed) with over sampling of the top and of the bottom, see text. This table provides some summary statistics on manual checks. The different possible outcomes are: "No info in sources" means the information is not included in Wikipedia/Wikidata nor in our dataset, "Info updated since data collection" means the information is included in Wikipedia/Wikidata but not present in our dataset; "Information Correct" means no error, "Error" means certain error, "Other case" means possible error (for instance historical controversy, several sources differing or information updated since data collection). These checks have been conducted by the 10 RAs and cross-verified by a PhD researcher.

Table S8. Manual verifications: summary statistics
Sample: top 1000 indiv.

	Birth	Death	Gender	1st Occupation	2nd Occupation	1st Citizenship	2nd Citizenship
Information not reported in our database							
No info. in sources	0.20	33.13	0.00	0.00	55.66	0.00	80.28
Info. updated since collec.	0,00	0.00	0.00	0.00	6.11	0.00	1.90
Information reported in our database							
Correct	98.00	66.17	100.00	100.00	36.54	99.30	16.32
Error	0.30	0.20	0.00	0.00	1.70	0.70	1.40
Other case (ambiguity or info updated since collec.)	1.50	0.50	0.00	0.00	0.00	0.00	0.10
TOTAL	1,000	1,000	1,000	1,000	1,000	1,000	1,000

Notes. Test sample on the top 1000 most notable of the database. This table provides some summary statistics on manual checks. The different possible outcomes are: "No info in sources" means the information is not included in Wikipedia/Wikidata nor in our dataset, "Info updated since data collection" means the information is included in Wikipedia/Wikidata but not present in our dataset; "Information Correct" means no error, "Error" means certain error, "Other case" means possible error (for instance historical controversy, several sources differing or information updated since data collection). These checks have been conducted by the 10 RAs and cross-verified by a PhD researcher.

Table S9. **Manual verifications: summary statistics**
Sample: 2+ Europ. `Wikipedia` eds.

	Birth	Death	Gender	1st Occupation	2nd Occupation	1st Citizenship	2nd Citizenship
Information not reported in our database							
No info in sources	3.80	56.80	0.00	0.00	66.40	0.00	91.80
Info. updated since collec.	0.40	0.60	0.00	0.20	0.40	0.40	1.20
Information reported in our database							
Correct	93.60	41.80	100.00	99.20	26.80	98.80	6.20
Error	0.60	0.20	0.00	0.60	6.00	0.80	0.60
Other case (ambiguity or info updated since collec.)	1.60	0.60	0.00	0.00	0.40	0.00	0.20
TOTAL	500	500	500	500	500	500	500

Notes. Test sample on the subset of the restricted database (at least **two** `Wikipedia` editions among the 7 European languages analyzed). This table provides some summary statistics on manual checks. The different possible outcomes are: “No info in sources” means the information is not included in `Wikipedia`/`Wikidata` nor in our dataset, “Info updated since data collection” means the information is included in `Wikipedia`/`Wikidata` but not present in our dataset; “Information Correct” means no error, “Error” means certain error, “Other case” means possible error (for instance historical controversy, several sources differing or information updated since data collection). These checks have been conducted by the 10 RAs and cross-verified by a PhD researcher.

Table S10. **Manual verifications: summary statistics**
Sample: `Wikidata` only, no `Wikipedia`

	Birth	Death	Gender	1st Occupation	2nd Occupation	1st Citizenship	2nd Citizenship
Information not reported in our database							
No info. in sources	70.20	80.80	20.40	35.00	96.00	60.60	99.40
Info. updated since collec.	4.40	1.60	33.60	24.40	1.40	4.80	0.20
Information reported in our database							
Correct	23.20	15.00	45.80	40.20	2.40	34.40	0.20
Error	0.00	0.00	0.00	0.20	0.00	0.00	0.00
Other case (ambiguity or info. updated since collec.)	2.20	2.60	0.20	0.20	0.20	0.20	0.20
TOTAL	500	500	500	500	500	500	500

Notes. Test sample on the those with **no** `Wikipedia` edition among the 7 European languages analyzed. This table provides some summary statistics on manual checks. The different possible outcomes are: “No info in sources” means the information is not included in `Wikipedia`/`Wikidata` nor in our dataset, “Info updated since data collection” means the information is included in `Wikipedia`/`Wikidata` but not present in our dataset; “Information Correct” means no error, “Error” means certain error, “Other case” means possible error (for instance historical controversy, several sources differing or information updated since data collection). These checks have been conducted by the 10 RAs and cross-verified by a PhD researcher.
